# Assessment of the public health risk of novel reassortant H3N3 avian influenza viruses that emerged in chickens

**DOI:** 10.1128/mbio.00677-25

**Published:** 2025-06-16

**Authors:** Han Li, Qi Tong, Mengyan Tao, Jixiang Li, Haili Yu, Qiqi Han, Jiancheng Wu, Riguo Lan, Jingjing Han, Haoyu Chang, Yan Li, Juan Pu, Yipeng Sun, Litao Liu, Yajin Qu, Quanlin Li, Lu Lu, Jinhua Liu, Honglei Sun

**Affiliations:** 1 National Key Laboratory of Veterinary Public Health and Safety, Key Laboratory for Prevention and Control of Avian Influenza and Other Major Poultry Diseases, Ministry of Agriculture and Rural Affairs, College of Veterinary Medicine, China Agricultural University 630101 , Beijing, Beijing, China; 2 The Roslin Institute, University of Edinburgh 15551, Edinburgh, Scotland, United Kingdom; University of Florida College of Public Health and Health Professions, Gainesville, Florida, USA

**Keywords:** H3N3, avian influenza viruses, genetic reassortment, public health

## Abstract

Influenza A (H3N2) viruses are historically responsible for the 1968 Hong Kong flu pandemic. Since then, H3N2 has continued to circulate as a seasonal influenza virus in humans. Public health concerns were raised in 2022 when human infections with novel reassortant H3N8 influenza viruses originating from chickens were first reported in China. Here, we conducted a systematic surveillance of H3 avian influenza viruses (AIVs) circulating in poultry and assessed the public health risk of emergent H3 reassortants. We found that H3 AIVs were prevalent in both ducks and chickens. Notably, in December 2022, a novel chicken-derived H3N3 subtype virus was identified, which gradually replaced the previously predominant H3N8 virus and became prevalent in chickens. Genetic analysis demonstrated that the novel H3N3 virus is a triple-reassortment strain with the H3 gene segment from chicken H3N8 virus, the N3 gene segment from the H10N3 virus, and internal gene segments derived from H9N2 viruses. Compared with chicken H3N8 and duck H3N3 viruses, the novel chicken H3N3 viruses produced higher yields and induced greater pathogenicity in human respiratory epithelial cells and mammalian models (mouse and ferret). Importantly, the chicken H3N3 viruses could be transmitted efficiently between ferrets through direct contact. The polymerase activity of the chicken H3N3 viruses in mammalian cells was markedly increased by the PA gene originating from the H9N2 virus. Our findings indicate that the circulation of novel chicken H3N3 viruses poses a threat to both the poultry industry and human public health.

**IMPORTANCE:**

The H3Ny subtype influenza A virus can infect a wide range of hosts. In addition to circulating among wild birds and poultry, the virus can also infect humans and a variety of mammals. Here, we found that H3Ny subtype AIVs were widely prevalent in domestic chickens and ducks. Novel H3N3 reassortant viruses emerged as a result of the genetic reassortment of the chicken-derived H3N8 AIVs with H10N3 and H9N2 AIVs. The novel H3N3 subtype AIVs are gradually displacing H3N8 AIVs and becoming prevalent in chickens. Furthermore, these novel H3N3 AIVs exhibited enhanced infection ability and efficient transmissibility in mammalian models, indicating a growing potential public health risk.

## INTRODUCTION

Avian influenza viruses (AIVs) pose a significant threat to wild birds, domestic poultry, and public health. Among various AIVs of different HA subtypes, H3 viruses could infect a broad range of hosts (1). H3 viruses not only circulate in wild waterfowl but are also common in domestic ducks and often reassort genetically with other AIV subtypes (2, 3). H3 viruses could occasionally spread to mammalian hosts, including humans, swine, canines, equines, cats, seals, and so on (4–8). A global pandemic was triggered in 1968 by a new H3N2 subtype virus that carried avian-derived hemagglutinin (HA) genes in Hong Kong (9). The pandemic H3N2 virus has since circulated in humans as a seasonal virus. Given the demonstrated potential of animal-derived H3 viruses to contribute to the emergence of novel reassortants with pandemic potential, continued surveillance and characterization of newly emerging variants are essential for mitigating future public health risks.

Ongoing AIV surveillance has been conducted in domestic poultry farms and live poultry markets (LPMs) in China. Notably, in 2021, a novel reassortant H3N8 subtype AIV was isolated from chicken flocks in southern China. The virus is a triple-reassortant strain with the Eurasian avian H3 gene, the North American avian N8 gene, and the internal genes from H9N2 viruses (10). Subsequently, the H3N8 viruses have spread rapidly to eastern and northern China (11–15). In 2022, the emerging H3N8 AIVs were implicated in two confirmed human infections, with a fatal case reported in 2023 (10, 16, 17). Biological characterization revealed that the novel H3N8 AIVs possess dual receptor-binding capability for both human and avian receptors and exhibit high pathogenicity in human respiratory epithelial cells, as well as in mouse and ferret models (10, 11, 15, 18–20). Importantly, the H3N8 virus isolated from a severe human case demonstrated efficient transmissibility in ferrets through respiratory droplets (20, 21). In addition, serological studies indicate that human sera lack cross-reactive immunity against the emerging avian H3N8 viruses (10–12, 16, 18, 21). Collectively, these findings highlight that the rapid genetic changes of the novel H3N8 viruses in poultry could pose an ongoing pandemic threat. Given these risks, continued surveillance of H3 AIVs in poultry, along with comprehensive risk assessments of emerging H3 variants, is crucial for public health preparedness.

In this study, we conducted an extensive AIV surveillance program across 13 provinces with high-density poultry populations from May 2022 to July 2023. We found that H3Ny subtype AIVs were widely prevalent in domestic ducks and chickens. Notably, H3N8 AIVs in chickens underwent further genetic reassortment with H10N3 and H9N2 AIVs, leading to the emergence of novel H3N3 reassortant viruses, which are becoming prevalent in chicken flocks. Furthermore, the novel H3N3 AIVs showed efficient infection ability and transmissibility in the mammalian models, indicating a rising public health threat.

## RESULTS

### Emergence and prevalence of H3N3 AIVs in chickens

To evaluate the prevalence of H3 AIVs in domestic poultry, 1,034 AIV strains were isolated from a total of 4,865 samples collected from LPMs and diseased poultry from May 2022 to July 2023, among which 212 samples belonging to H3Ny subtype viruses were identified. Among them, 136 isolates were from chickens, and 76 isolates were from ducks (Table S1). Duck-derived H3 viruses were identified with multiple neuraminidase (NA) subtypes: N1 (*n* = 7), N2 (*n* = 53), N3 (*n* = 2), N6 (*n* = 7), and N8 (*n* = 7), and most duck-derived H3 AIVs (64/76, 84.21%) were detected from apparently healthy ducks in LPMs. Meanwhile, chicken-derived H3 viruses were only identified as two NA subtypes: N8 (*n* = 91) and N3 (*n* = 45). Importantly, we first detected the H3N3 subtype virus in chickens in December 2022, which initially caused an outbreak of respiratory disease among broilers in the Yangtze River Delta. The epidemic subsequently expanded to eastern and northern China. Since 2023, the detection rate of H3N3 has steadily increased, while that of the H3N8 virus has declined, suggesting that the H3N3 virus is gradually becoming predominant in the poultry population (Fig. S1). It is worth noting that 80% (36/45) of H3N3 viruses were isolated from laying hens, which exhibited a 10–40% decrease in egg production, indicating that H3N3 AIV infection is emerging as a significant disease in layer hens. Antigenic analysis further indicated that chicken- and duck-derived H3 AIVs belonged to different antigenic groups, whereas chicken H3N3 and H3N8 viruses belonged to the same antigenic group (Fig. S2). These findings highlight the rapid emergence and dominance of H3N3 AIVs in chickens.

### Chicken H3N3 viruses exhibit abundant genetic diversity

**Fig 1:**
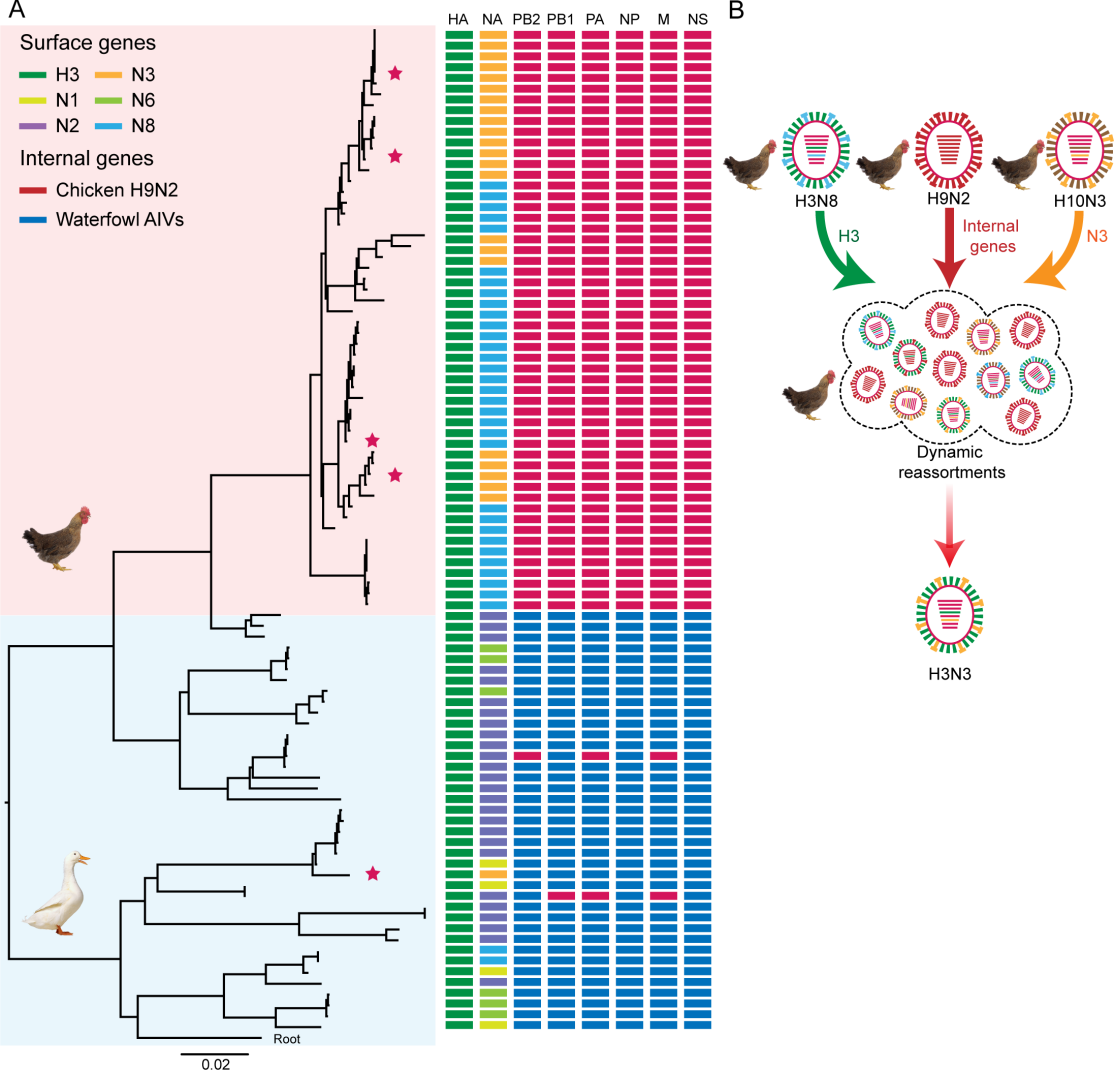
Phylogenetic analysis of H3Ny subtype avian influenza viruses (AIVs). (A) Phylogenetic tree of the hemagglutinin (HA) gene and genotype evolution of H3 AIVs. The phylogenetic tree was reconstructed using genetic distances calculated by maximum likelihood under the general time-reversible model. A detailed phylogenetic tree with sequence names is shown in Fig. S2. The pink block indicates chicken sublineage, while the light green block indicates duck sublineage. The strains used for biological characterization were indicated by asterisks. Colored boxes show the lineage classification for each gene segment of H3Ny viruses. (**B**) Schematic representation of the origins and dynamic reassortments of the novel H3N3 virus. The eight gene segments (i.e., horizontal bars) are PB2, PB1, PA, HA, NP, NA, M, and NS, from top to bottom.

To understand the evolution history of H3 viruses, we selected 90 representative H3 isolates according to the collection date, location, and host for full genome sequencing. Phylogenetic analysis of the HA gene revealed that the duck-derived H3 viruses exhibited a relatively low identity (ranging from 79.6 to 100%) in the HA gene and formed multiple distinct subclades. In contrast, chicken-derived H3 viruses clustered into a single phylogenetic subclade with strong bootstrap support (100%) and shared a higher sequence identity (96.1–100%) (Fig. 1A). Phylogenetic analysis of NA genes showed that the N1, N2, N3, and N6 genes from duck-derived H3 viruses belonged to the Eurasian avian lineage. The N3 genes of chicken-derived H3N3 viruses fell into the Eurasian avian lineage and shared a high sequence identity (>96.7%) with those of the H10N3 virus, which had been prevalent in chickens in the Yangtze River Delta and had caused four documented human infections since 2021 (22–25). The N8 genes of the chicken H3N8 virus fell within the North American lineage and formed a distinct subclade with a bootstrap support value of 100% and also demonstrated a high homology (sequence identity > 96.7%) (Fig. S3). The six internal genes of chicken-derived H3Ny isolates originated from G57 genotype H9N2 viruses, while the duck-derived H3Ny viruses mainly originated from Eurasian waterfowl AIVs (Fig. 1 and Fig. S3). It is noted that the internal genes of chicken-derived H3N3 viruses could be grouped into multiple clusters, which were closely related to those of the H9N2, H3N8, or H10N3 viruses prevalent in the same province (Fig. S3). These results suggest that the H9N2 virus gene pools contribute to the increasing genetic diversity of the H3N3 virus through dynamic reassortment (Fig. 1B). Overall, the H3N8 virus underwent dynamic reassortment with H10N3 and H9N2 viruses during the epidemic period, leading to the emergence of the genetically diverse H3N3 virus.

### Chicken H3N3 viruses exhibit abundant genetic markers for mammalian host adaptation

We analyzed the key molecular markers in the 90 H3 AIV genomes (Tables S4 and S5). All of the chicken H3N3 and duck H3Ny viruses contained two basic amino acids at the cleavage site of HA, implying that these strains exhibit low pathogenicity in chickens. Notably, seven chicken H3N8 isolates possessed three basic amino acids (motif: PEKQKR/GLF), which might enhance the viral pathogenicity in poultry. Key residues in the receptor-binding site (RBS) revealed that all the chicken H3Ny viruses maintained the avian signature Q226 and G228 in the 220-loop of the HA gene. Meanwhile, these viruses also possessed residues N193, W222, and S227 in the RBS, conferring the viruses with human and avian dual receptor-binding properties. Analysis of the NA gene showed that none of the chicken H3Ny viruses had a stalk-deletion variant, which was associated with the adaptation of AIVs to terrestrial poultry (26, 27). The six internal genes of the chicken H3Ny viruses were all derived from H9N2 viruses, which had accumulated a large number of mammalian-adaptive mutations. Although the well-known E627K mutation of the PB2 gene was not found in chicken H3Ny viruses, several other mammalian-adaptive mutations were detected. These included A37S, K356R, and S409N mutations in the PA gene, which could enhance polymerase activity, as well as the V15I mutation in the M1 gene and the E227K mutation in the NS1 gene, both of which have been linked to enhanced pathogenicity in mammalian hosts (28). This indicates that the chicken H3N3 viruses acquired multiple mammalian-adaptive mutations, which may enhance their adaptability to mammalian hosts and facilitate cross-species transmission.

### Chicken H3N3 viruses replicate efficiently in human airway epithelial cells

**Fig 2:**
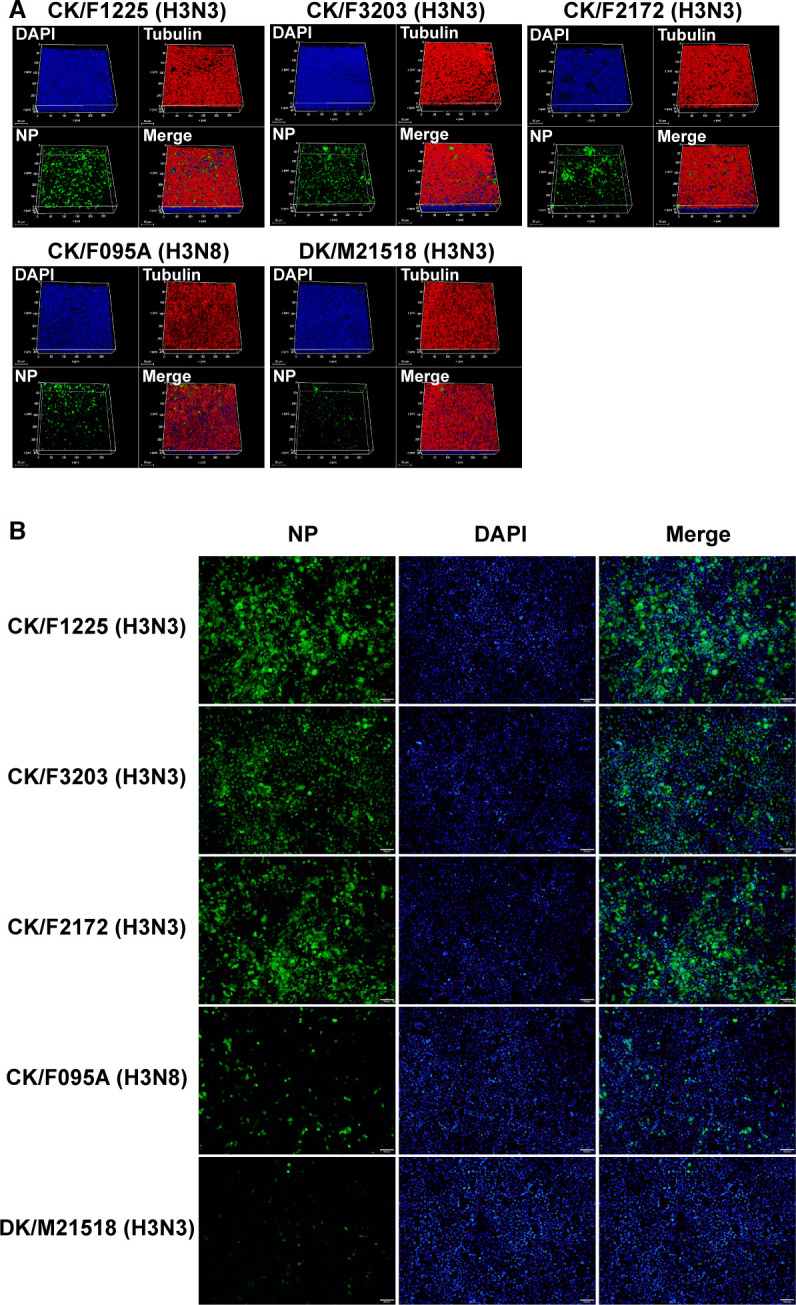
Infection of H3Ny viruses in organotypic normal human bronchial epithelial (NHBE) cultures and Calu-3 cells. (A) Three-dimensional (z stack) imaging of H3Ny subtype viruses in NHBE cultures. NHBE cells were infected with each virus at 1.0 multiplicity of infection (MOI) for 12 h, and viral NP (green) was detected by indirect immunofluorescence. Ciliated epithelial cells were detected with anti-β-tubulin IV antibodies (red), and nuclei were immunostained with 4′,6-diamidino-2-phenylindole (DAPI) (blue). A series of confocal images was taken at a magnification of ×40 from the stained piece of epithelium at a distance of the *Z* value (in micrometers) shown in each image from the objective (*z* axis) and reconstituted as a three-dimensional (z stack) image as shown in each channel of fluorescence. (**B**) Calu-3 cells were infected with the indicated viruses at an MOI of 1.0. Influenza virus NP was detected at 12 hpi with an fluorescein-isothiocyanate (FITC)-conjugated goat anti-mouse antibody (left column). Nuclei were detected with DAPI (middle column). Merged channels are displayed on the right column. Scale bars, 100 µm.

Given the prevalence of the emerging H3N3 virus in chickens, it is necessary to assess its potential risk of human infection. We selected three chicken H3N3 viruses (A/chicken/Shandong/F3203/2023 [CK/F3203], A/chicken/Jiangsu/F1225/2022 [CK/F1225], and A/chicken/Anhui/F2172/2023 [CK/F2172]), one duck H3N3 virus (A/duck/Jiangxi/M21518/2023 [DK/M21518]), and one chicken H3N8 virus (A/chicken/Henan/F095A/2022 [CK/F095A]) for further biological characterization. We first inoculated the representative virus strains at a multiplicity of infection (MOI) of 1.0 in normal human bronchial epithelial (NHBE) cells and examined the percentage of infected cells by using immunofluorescence at 12 h post-infection (hpi). As shown in Fig. 2A, the number of cells infected with chicken H3N3 viruses (>15%) was significantly higher (*P* < 0.0001) than that of cells infected with the chicken H3N8 virus (6.9 ± 1.6%) and the duck H3N3 virus (1.0 ± 0.43%) (Fig. S4A). Similar results were observed in the corresponding human lung epithelial (Calu-3) cells (Fig. 2B and Fig. S4B). Through scanning electron microscopy, the chicken H3N3 virus was found to cause more severe morphological damage and cilia exfoliation in NHBE cells (Fig. S4E).

Fully differentiated NHBE and Calu-3 cells were separately infected at 0.01 MOI. Virus titers were determined subsequently. The chicken H3N3 strains grew to comparable levels in NHBE cells at each time point and reached peak titers of around 10^6.19^ 50% tissue culture infectious dose (TCID_50_)/mL at 48 hpi. In contrast, the chicken H3N8 virus (CK/F095A) and the duck H3N3 virus (DK/M21518) exhibited peak titers of 10^3.75^ and 10^2.5^ TCID_50_/mL, respectively (Fig. S4C). In Calu-3 cells, from 12 h of infection onward, the chicken H3N3 viruses produced more viable progeny particles than both the chicken H3N8 and duck H3N3 viruses. At peak replication, the mean viral titers of the chicken H3N3 viruses were 100–1,000 times higher than those of duck H3N3 viruses (Fig. S4D). Collectively, these findings indicated that compared to chicken H3N8 and duck H3N3 viruses, chicken H3N3 viruses exhibit enhanced infection and replication abilities in human respiratory epithelial cells.

### Chicken H3N3 viruses elicit enhanced replication ability in mice and ferrets

**Fig 3:**
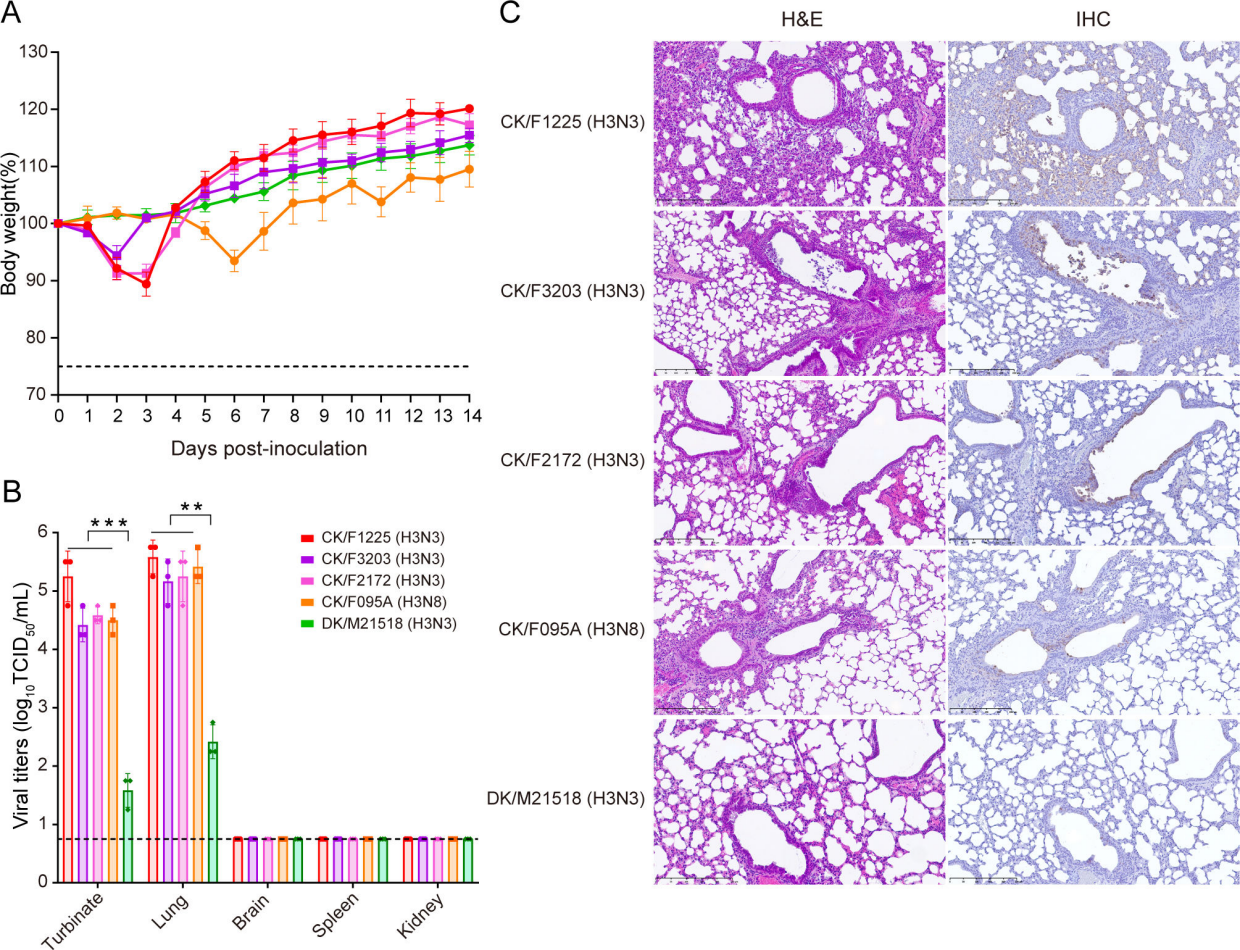
Pathogenicity and replication of H3Ny subtype viruses in mice. (A) Groups of five mice were i.n. infected with the indicated viruses at doses of 10^6^ TCID_50_, and body weight changes were monitored for 14 days. The dashed line indicates 75% of the initial body weight. (**B**) Mice were infected at doses of 10^6^ TCID_50_ of the indicated viruses, and three mice per group were euthanized at 4 dpi. Virus titers in organs (turbinate, lung, brain, spleen, and kidney) were determined by TCID_50_ assay on the Madin-Darby canine kidney (MDCK) cells. Data are shown as mean ± SD. Statistical significance was assessed by using one-way analysis of variance compared with the corresponding value of duck H3N3 virus DK/M21518 (**P < 0.01, ***P < 0.001). Dashed lines indicate the lower limit of detection. (**C**) Representative histopathological findings in the lungs of mice infected with the indicated viruses at 4 dpi. Lung sections were stained with hematoxylin and eosin (H&E) (left) and by immunohistochemistry (IHC) against influenza viral NP antigen (right). Scale bars, 250 µm.

To evaluate the pathogenicity and replication ability of H3N3 viruses in mammalian hosts, groups of eight 6-week-old female BALB/c mice were intranasally (i.n.) inoculated with 50 µL of 10^6^ TCID_50_ of each virus and monitored daily over 14 days. We found that all the mice survived during the 14-day observation period. Mice infected with chicken H3N3 viruses developed weight loss after inoculation, while no clinical signs or body weight reduction was observed in the duck H3N3 virus-infected group (Fig. 3A). To assess *in vivo* virus replication, the turbinate, lung, brain, spleen, and kidney tissues were collected for virus titration at 4 days post-infection (dpi). All chicken H3N3 viruses replicated efficiently in the turbinates and lungs and displayed significantly higher virus titers than the duck H3N3 virus (*P* < 0.01) (Fig. 3B). Microscopically, peribronchiolitis and bronchopneumonia were found in the lungs of mice infected with chicken H3N3 viruses but not in those infected with the duck H3N3 virus. Immunohistochemistry (IHC) staining detected more viral antigen-positive cells in the lungs of mice infected with chicken H3N3 viruses (Fig. 3C).

**Fig 4:**
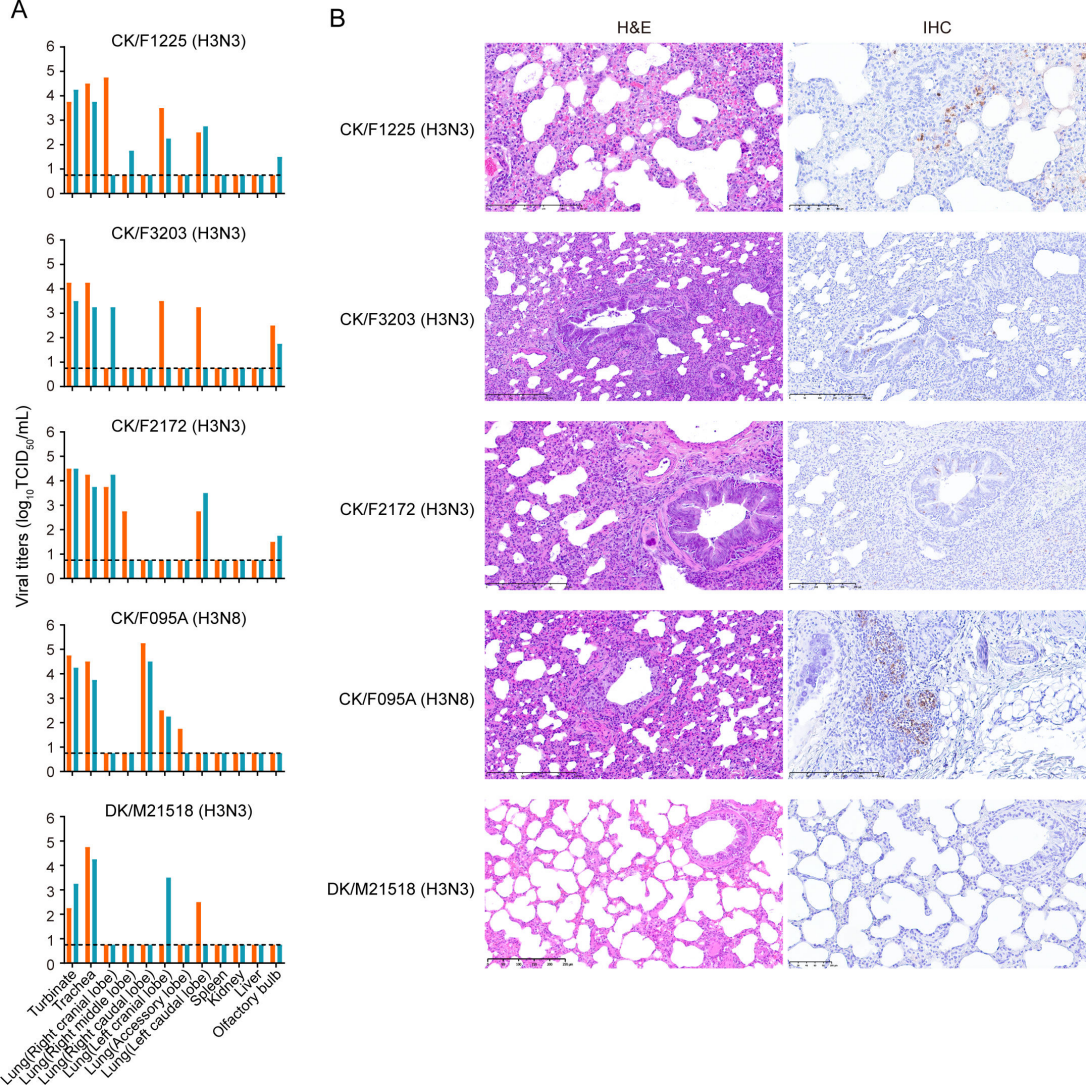
Virus infection and histopathology of ferrets infected with H3Ny subtype viruses. Two ferrets per group were infected i.n. with 10^6^ TCID_50_ of the indicated viruses. (**A**) Ferrets were euthanized at 4 dpi, and tissues were taken for virus titration by TCID_50_ assays on the MDCK cells. Dashed lines indicate the lower limit of detection. (**B**) Representative lung pathological findings at 4 dpi. Lung sections were stained with H&E (left) and by IHC against influenza viral NP antigen (right). Scale bars, 250 µm.

Ferrets (*Mustela putorius furo*) are ideal animal models to consider when studying influenza virus infection, as they exhibit clinical symptoms similar to those observed in humans (29). Groups of two ferrets were inoculated i.n. with 10^6^ TCID_50_ of each virus. Ferrets inoculated with the duck H3N3 virus exhibited no apparent clinical manifestations. In contrast, ferrets inoculated with chicken H3N3 and H3N8 viruses had a transient temperature elevation and exhibited clear clinical signs, including wheezing and coughing. At 4 dpi, all ferrets were euthanized, and tissues were collected for virus titration and histopathological examination. We found that the chicken H3N3 viruses replicated much more efficiently than the duck H3N3 virus in the nasal turbinate, trachea, and lung in ferrets. Notably, chicken H3N3 viruses were detected in the olfactory bulb of infected ferrets (Fig. 4A). Chicken H3N3 viruses-infected ferrets exhibited bronchopneumonia in the lungs. Moreover, viral antigen-positive cells were more abundant in the lungs of ferrets infected with the chicken H3N3 virus (Fig. 4B). Collectively, these results indicate the chicken H3N3 viruses replicate efficiently in both mice and ferrets.

### Chicken H3N3 viruses efficiently transmit among ferrets by direct contact

**Fig 5:**
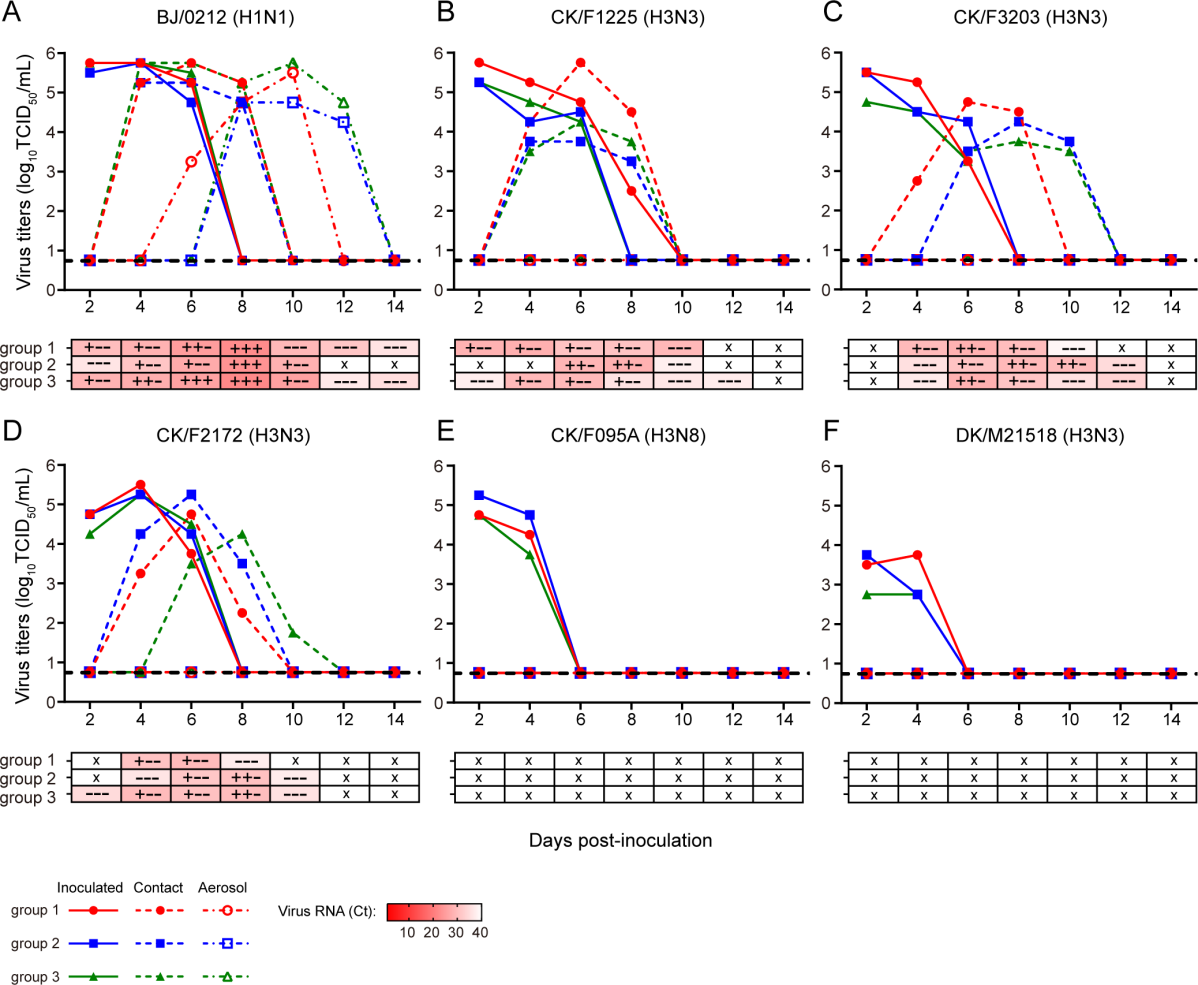
Horizontal transmission of H3Ny subtype viruses between ferrets. One ferret was anesthetized and i.n. inoculated with 10^6^ TCID_50_ of BJ/0212 (A), CK/F1225 (B), CK/F3203 (C), CK/F2172 (D), CK/F095A (E) or DK/M21518 (F). The following day, the inoculated ferret was individually paired by cohousing with a DC ferret; an RD-contact ferret was also housed in a wire frame cage adjacent to the infected ferret. Three independent replicates were conducted for each virus group. Nasal washes for virus shedding detection were collected every other day from all animals from 2 days after the initial infection. Virus titers were determined by TCID_50_ assays on MDCK cells. Each color bar represents the virus titers of an individual animal. Dashed lines indicate the lower limit of virus detection. Viral RNA loads in air samples were quantified by quantitative reverse transcription polymerase chain reaction (qRT-PCR) targeting the AIV M gene, and the heatmap represents the Ct value of each sample. The × indicates “no Ct” value below the positive cutoff. Live virions of the air samples were detected using specific pathogen-free chicken embryos. The + indicates a positive result of viral culture in one chicken embryo, and the − represents a negative result.

To assess the transmissibility of H3N3 viruses, we performed direct-contact (DC) and respiratory-droplet (RD) virus transmission experiments on ferrets. Human pandemic H1N1 (pdm/09 H1N1) virus (A/Beijing/0212/2019 [BJ/0212]) was used as a positive control. Briefly, ferrets (*n* = 3) were inoculated i.n. with 10^6^ TCID_50_ of each virus. At 24 hpi, infected ferrets were individually paired by cohousing with a DC ferret. An RD-contact ferret was also housed in a wire frame cage adjacent to an infected ferret. Nasal washes for virus shedding detection were collected every other day starting at 2 dpi, and seroconversion was determined at 21 dpi. As expected, human H1N1 virus was transmitted to all ferrets via DC on 4 dpi, while RD transmission was detected on 6 dpi (Fig. 5A). In the chicken H3N3 virus groups, viruses were detected in nasal washes of all directly infected ferrets from 2 to 6 dpi and all DC animals from 4 to 10 dpi (Fig. 5B through D), whereas no RD spread was detected. Seroconversion of DC animals further corroborated contact transmission (Table S3). In contrast, in the chicken H3N8 and duck H3N3 virus groups, viral detection was limited in the nasal washes of directly infected animals from 2 to 4 dpi, with no evidence of contact or airborne transmission (Fig. 5E and F). These results indicate that chicken H3N3 viruses efficiently transmit among ferrets by direct contacts, whereas H3N8 viruses do not.

To further understand the airborne virus shedding kinetics, air samples were collected every other day for 14 days in all transmission experiments by using samplers capable of collecting inhalable bioaerosols. Air samples were quantified by quantitative reverse transcription polymerase chain reaction (qRT-PCR) for viral RNA and inoculated into chicken eggs for live viral particles. In the human H1N1 virus (BJ/0212) group, all three groups of infected ferrets consistently released influenza viral RNA, reaching a mean peak of 5.3 log_10_ RNA copies/mL on day 8, and infectious virus was detected from 2 to 10 dpi (Fig. 5A). In chicken H3N3 groups, the ferrets continuously shed viral RNA from 2 to 10 dpi, with a mean peak of 3.2 log_10_ RNA copies/mL, and the infectious virus was detected in the air during the same period (Fig. 5B through D). In contrast, no viral RNA or infectious virus was detected in the air samples collected from chicken H3N8 and duck H3N3 virus groups at any time point post-infection. Overall, these data reveal that chicken H3N3 viruses not only replicate in the respiratory tract of ferrets but also shed into the air, indicating a potential public health risk posed by the novel H3N3 viruses.

### PA gene significantly enhances the viral polymerase activity of chicken H3N3 viruses in mammalian cells

**Fig 6:**
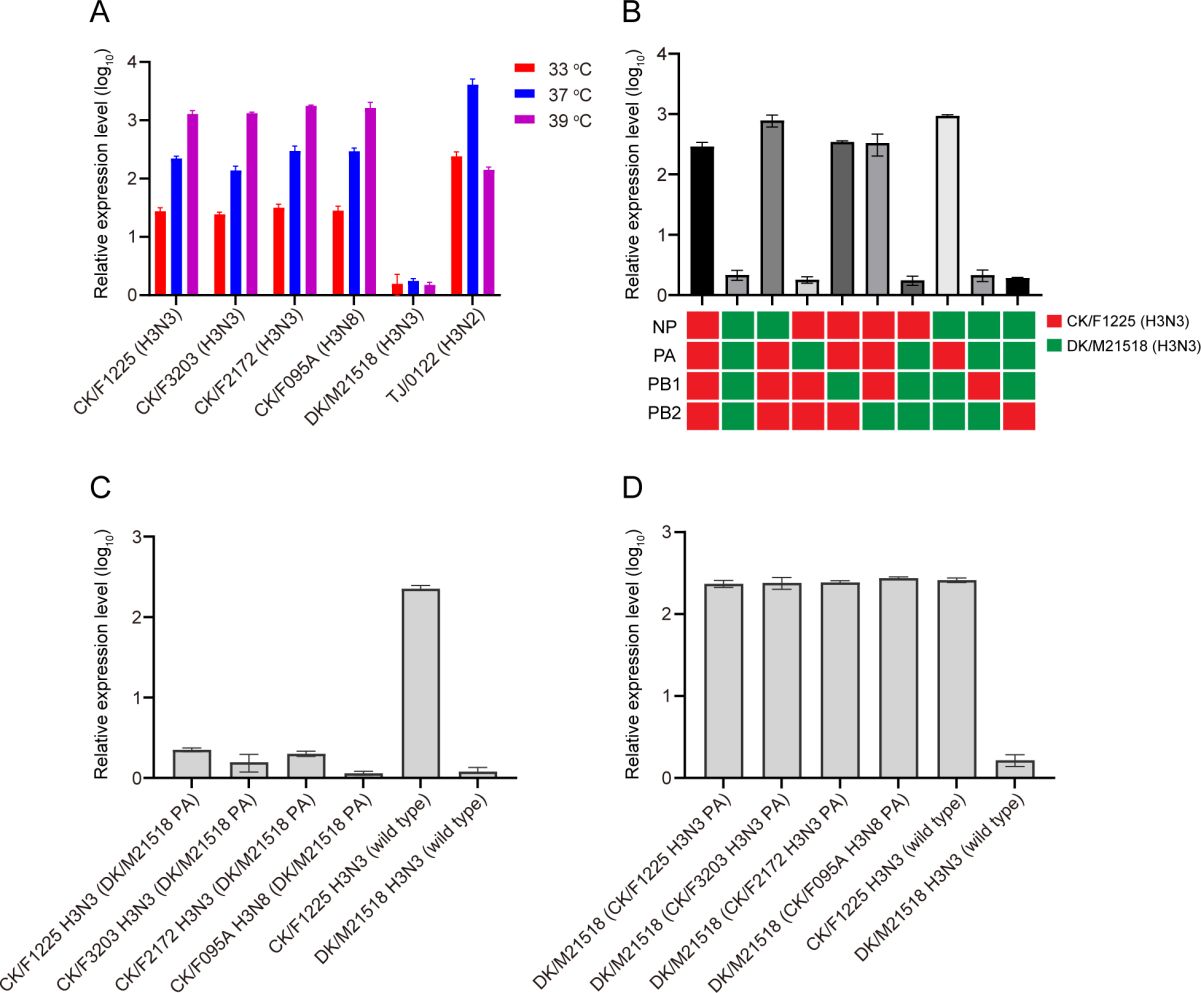
Polymerase activity of H3Ny subtype viruses. (A) Polymerase activity of the vRNP complex of H3 viruses. Human embryonic kidney 293T (HEK293T) cells were transfected with luciferase reporter plasmid p-Luci and internal control plasmid Renilla, together with plasmids expressing PB2, PB1, PA, and NP from the H3 viruses. Cells were incubated at 33, 37, or 39°C for 24 h, and cell lysates were analyzed to measure firefly and Renilla luciferase activities. Values represent the mean ± SD of the three independent experiments. (**B**) Polymerase activity of 10 vRNP combinations between the chicken- and duck-derived H3N3 viruses at 37°C. (**C**) Duck-derived H3N3 virus PA gene was individually introduced into the chicken-derived H3 virus vRNP and tested for polymerase activity at 37°C. (**D**) Chicken-derived H3Ny virus PA gene was individually introduced into the duck-derived H3N3 virus vRNP and tested for polymerase activity at 37°C.

Polymerase activity is a key indicator of the replicability and pathogenicity of AIVs. We quantified polymerase activities of chicken H3N3 (CK/F1225), chicken H3N8 (CK/F095A), and duck H3N3 (DK/M21518) strains in human embryonic kidney 293T (HEK293T) cells at temperatures mimicking the upper (33°C) and lower (37 and 39°C) respiratory tract microenvironments. Human H3N2 virus (A/Tianjin/0122/2018 [TJ/0122]) was used as control. As shown in Fig. 6A, chicken-derived H3N3 and H3N8 viruses demonstrated significantly higher luciferase activity than the duck-derived H3N3 virus at all tested temperatures (*P* < 0.0001), with maximal enhancement observed at physiological 37°C (125- and 166-fold increases for chicken H3N3 and H3N8, respectively). To identify the genetic basis for enhanced polymerase activity in chicken H3Ny viruses, we generated reassorted polymerase complexes using chicken-derived H3N3 (CK/F1225) and duck-derived H3N3 (DK/M21518) genes, then assessed their polymerase activity at 37°C. When the chicken-derived H3N3 PA gene (H9N2 virus-derived) was integrated into the duck-derived H3N3 RNP complexes, the polymerase activity increased to the levels comparable to those of the chicken H3N3 RNP complex (Fig. 6B). Conversely, the complexes containing the duck-derived H3N3 PA gene showed drastically reduced polymerase activity similar to that of the duck-derived H3N3 RNP complex (Fig. 6B). To further confirm the essential role of the H9N2 virus-derived PA gene in polymerase activities, we introduced the duck H3N3 virus-derived PA gene to the chicken H3Ny viruses, resulting in a significant reduction in polymerase activity in all recombinant strains (Fig. 6C). Conversely, replacing the PA gene in the duck H3N3 virus with that from the chicken H3Ny viruses, all the recombinant strains showed a significant increase in polymerase activity (Fig. 6D). Our results demonstrate that the H9N2 virus-derived PA gene could significantly enhance the viral polymerase activity of chicken H3N3 viruses in mammalian cells, which could potentially promote the adaptation of the H3N3 virus to mammalian hosts.

## DISCUSSION

The emergence of avian-derived H3N8 influenza viruses in 2022 and their ability to infect humans raised significant concerns about the pandemic potential of emerging H3Ny viruses (5, 11–13). Given the ongoing evolution of these viruses, continuous surveillance in poultry and comprehensive risk assessments of emerging H3 reassortants are critical for early detection and mitigation of potential public health threats. In this study, we revealed that H3N8 viruses circulating in chicken flocks have undergone frequent reassortment with H9N2 and H10N3 viruses, resulting in the emergence of novel H3N3 viruses, and the H3N3 viruses are gradually replacing H3N8 viruses and becoming prevalent in chickens. The internal genes derived from H9N2 viruses, especially the PA gene, significantly enhance the polymerase activity of chicken H3Ny virus in mammalian cells. Notably, compared to H3N8, the novel H3N3 virus exhibited increased viral shedding and efficient contact transmission in ferrets, indicating further adaptation to mammals.

Genetic reassortment among influenza viruses drives viral evolution and facilitates the emergence of novel strains with pandemic potential. Our previous research demonstrated that H3N8 viruses had been generated by reassortment events involving the H3 gene from domestic waterfowl, the N8 gene from wild aquatic birds, and internal genes from chicken-derived H9N2 viruses. Subsequently, these H3N8 viruses continued undergoing genetic recombination with locally prevalent H9N2 viruses (known as G57 genotype) in chicken populations (10). Then, the novel H3N3 viruses emerged through reassortment of H3N8, H10N3, and G57 genotype H9N2 viruses, which were prevalent in chicken flocks in the Yangtze River Delta. The predominant G57 genotype H9N2 AIVs in chickens since 2010 have been the most prevalent AIV subtype among chicken flocks in China (30, 31). However, Pekin and mallard ducks have natural resistance to H9N2 virus infection, which makes it difficult for the virus to effectively infect and replicate via the respiratory infection route (32). Therefore, H9N2 viruses circulate in terrestrial poultry rather than waterfowl, indicating that the genetic reassortment of H9N2 with other subtypes of AIVs mainly occurs in chicken flocks. These novel reassortant AIVs frequently spill over to humans, as evidenced by infections with H3N8 (10), H5N6 (33), H7N9 (34), H10N8 (35), and H10N3 (22). This phenomenon is associated with the accumulation of multiple mammalian-adaptive mutations in the H9N2 viruses, which enhances the cross-species transmission ability of novel reassortant viruses containing the internal genes from H9N2 subtype AIVs (28).

In this study, we found that vRNPs originating from H9N2 virus-derived internal genes possess higher polymerase activity, with the PA gene having the most significant impact. The PA protein functioning as both an endonuclease and a protease participates in the binding of the viral RNA and complementary RNA (cRNA) promoter, can be transferred into nuclei using nuclear localization signals, and regulates cRNA and viral RNA synthesis (36). The molecular mechanisms underlying how the PA gene derived from H9N2 viruses enhances polymerase activity still require intensive study.

Notably, H10N3 subtype AIVs have circulated widely in chicken flocks in the Yangtze River Delta and have caused four human infections since 2021 (22–25). The NA protein, a sialidase, is responsible for cleaving the sialic acid receptors from galactose. The balance between the activities of HA and NA (HA-NA balance) is crucial for efficient replication, pathogenicity, transmissibility, and cross-species transmission of influenza viruses (37). Here, we found that chicken-derived H3N3 viruses displayed increased viral shedding and efficient direct-contact transmission in ferrets compared with chicken-derived H3N8 viruses. This suggests that the H3N3 virus has achieved a functional HA-NA balance that enhances its adaptation for mammalian hosts.

The ferret transmission model serves as a gold standard for assessing the human transmissibility of emerging AIVs (38). Our analysis showed the kinetics of virus shedding following infection and quantified the viral load released into the air. We found that while the viral titers were similar in nasal washes of ferrets infected with H3N3 and H3N8 viruses, only the H3N3-infected ferrets could shed live viral particles into the air. Although airborne transmission of the H3N3 virus between ferrets was not detected in this study, the ability of these viruses to cause infection through direct contact and shed live viral particles into the environment deserves attention. Once the H3N3 virus acquires critical mutations, the risk of human infection among poultry farm workers and individuals in live poultry markets could substantially increase. Therefore, incorporating environmental detection of viruses into the frame of risk assessment can provide a more objective and accurate evaluation of the transmission risks posed by novel influenza virus.

In summary, our study revealed the emergence and increasing prevalence of H3N3 viruses in chicken populations, highlighting their enhanced transmissibility and pathogenicity in human respiratory epithelial cells and mammalian models compared to chicken H3N8 viruses. Notably, H3N3 viruses demonstrated efficient direct-contact transmission in ferrets, indicating their potential for cross-species transmission. Collectively, these findings suggest that H3N3 viruses might pose a risk to public health, emphasizing the urgent need for comprehensive surveillance and control of the spread of H3Ny reassortant viruses in poultry.

## MATERIALS AND METHODS

### Cells

MDCK cells, HEK293T, and Calu-3 cells were cultured with Dulbecco’s modified Eagle medium (DMEM; Gibco, New York, NY, USA) supplemented with 10% fetal bovine serum (Gibco, Sydney, Australia) and 1% penicillin-streptomycin solution (MACGENE, Beijing, China). NHBE cells were purchased from American Type Culture Collection. NHBE cells were thawed, seeded at 5,000 cells/cm^2^, and expanded in PneumaCult-Ex Plus Medium (STEMCELL Technologies, Vancouver, Canada). Cells of passage 2 were grown on 12-well Transwell inserts (Corning, Beijing, China) on the apical surface in PneumaCult-ALI Medium (STEMCELL Technologies, Vancouver, Canada). Full differentiation for experimental use took around 21 days of culture.

### Virus isolation and identification

We performed an extensive AIV surveillance program in 13 provinces with high-density poultry populations (i.e., Anhui, Guangdong, Fujian, Hebei, Henan, Jiangsu, Jiangxi, Zhejiang, Liaoning, Ningxia, Shandong, Guizhou, and Chongqing) from May 2022 to July 2023. A total of 4,166 nasal swabs were collected from apparently healthy poultry in LPMs, and a total of 699 tracheal or lung tissues were collected from diseased poultry (Table S1). Samples were diagnosed by the Key Laboratory for Prevention and Control of Avian Influenza and Other Major Poultry Diseases, Ministry of Agriculture and Rural Affairs, China. Virus isolation and the identification of HA and NA subtypes were performed as previously described (31). AIV-positive allantoic fluids were harvested and stored at −80°C.

### Genomic sequencing of H3Ny subtype viruses

Ninety representative H3Ny subtype strains were selected for sequencing based on virus collection date, location, and host, including at least one strain per province. Whole genomic DNA was sequenced by next-generation sequencing on an Illumina NovaSeq platform according to standard protocols. All nucleotide sequences of segments of virus isolates detected in this study have been deposited in the Global Initiative on Sharing Avian Influenza Data database (GISAID).

### Phylogenetic analyses

Complete genomes of AIVs for reference were downloaded from GISAID (http://www.gisaid.org). The genome sequences of the avian H3 viruses were compared with all the publicly available sequences as of August 2023. The resulting alignments were manually adjusted for subsequent phylogenetic analysis. The phylogenetic trees of each genomic segment were inferred based on the maximum likelihood method by FastTree 2.1.11 under the general time-reversible nucleotide substitution model, applying 1,000 bootstrap replicates.

### Antigenic analyses

Antigenic characteristics of H3Ny subtype viruses were compared with chicken antisera raised against representative viruses by hemagglutination inhibition (HI) assay. HI assays were conducted in V-bottom 96-well reaction plates with 1% chicken red blood cells. HI tests were performed in accordance with World Health Organization guidelines, and all HI assays were performed in duplicate. Analysis of antigenic properties was performed using antigenic cartography methods described previously (31), and the HI data were log_2_-transformed and downscaled to three-dimensional space by multidimensional scaling. Antigenic clustering of virulent strains was performed using K-means clustering.

### Immunofluorescence microscopy

The apical surface of NHBE cells was gently washed three times with warm Dulbecco’s phosphate-buffered saline (DPBS; MACGENE, Beijing, China) to remove accumulated mucus prior to infection. Calu-3 and NHBE cells were infected with the indicated viruses at 1.0 MOI. The inoculum was removed after 2 h, and cells were further incubated at 37°C. Cultures were fixed in 4% formaldehyde (Beyotime, Beijing, China) at 12 hpi. After 30 min of fixation, the cultures were permeabilized with 0.5% Triton X-100 (Beyotime, Beijing, China) for 30 min, incubated with blocking buffer (Beyotime, Beijing, China) for 30 min at 37°C, and immunostained with mouse anti-NP IgG antibody (1:5,000; ab20343, Abcam, Beijing, China) at 4°C overnight. Cultures were then incubated with fluorescein isothiocyanate-labeled goat anti-mouse IgG antibody (1:400; 5230-0427, KPL, Gaithersburg, USA). For localization of ciliated cells, fixed NHBE cultures were co-immunostained with anti-β tubulin IV antibody (1:1,000; ab179504, Abcam, Beijing, China) and Alexa Fluor 488-labeled goat anti-rabbit IgG antibody (1:1,000; A-11012, Life Technologies, Carlsbad, USA) for detection. NHBE cultures were imaged using the Leica TCS SP8 confocal laser scanning microscope (Leica, Wetzlar, Germany). A set of confocal images was taken at a magnification of ×40 from the stained piece of epithelium at a distance of the *Z* value (in micrometers), shown in each image, from the objective (*z* axis) and reconstituted as a three-dimensional (z stack) image. To count percentages of influenza NP-positive cells per DAPI-positive cells, maximum intensity projection was performed, and positive cells were quantified using ImageJ software (NIH public domain).

### Growth kinetics of viruses in NHBE and Calu-3 cells

The apical surface of NHBE cells was gently washed three times with warm DPBS to remove accumulated mucus prior to infection. Following 1 day post-washing, NHBE cultures were inoculated in triplicate with each virus at an MOI of 0.01. Inoculated virus was removed after 2 h of incubation and washed three times with DPBS. Each sample was harvested by the addition of 150 μL DMEM to the apical surface for 30 min at 37°C. Supernatants were sampled at 12, 24, 36, 48, 60, and 72 hpi and titrated by TCID_50_ on MDCK cells. The Calu-3 cells were infected with indicated viruses at an MOI of 0.01. After a 2 h incubation period, the cells were washed three times with DPBS and incubated at 37°C in 5% CO_2_. Supernatants were collected at 12, 24, 36, 48, 60, and 72 hpi, and viral titers were determined by TCID_50_ assays.

### Scanning electron microscopy

Scanning electron microscopy was performed as previously described. Briefly, NHBE cultures were infected with each virus at an MOI of 0.01. At 48 hpi, infected or mock NHBE cultures were fixed with 2.5% glutaraldehyde (Soliabio, Beijing, China) at 4°C overnight, washed and dehydrated in gradient ethanol, critical point-dried, and vacuum-evaporated. The membrane with NHBE cells was removed from the Transwell rack and glued to a sample holder for scanning electron microscopy (Hitachi SU8000, Tokyo, Japan).

### Mouse challenge studies

A total of 25 6-week-old female BALB/c mice (*n* = 5 per group) were anesthetized with Zoletil 50 (tiletamine-zolazepam [Virbac, Carros, France], 20 mg/g body weight) and i.n. inoculated with 50 μL virus at a dose of 10^6^ TCID_50_. The mice in each group were monitored daily for 14 days for weight loss. Mice that lost >25% of their initial body weight were euthanized. To monitor virus production, a total of 15 mice (*n* = 3 per group) infected with 10^6^ TCID_50_ virus were euthanized at 4 dpi. Nasal turbinates, right lung lobes, brain, spleen, and kidney tissues were collected for virus titration. The left lung tissue of each mouse was used for histology and IHC.

### Ferret challenge studies

Six-month-old male Angora ferrets (*M. putorius furo*) serologically negative for currently circulating influenza viruses (H1, H3, H5, H7, and H9) and >1.0 kg (ranging from 1.10 to 1.80 kg) in weight were purchased from Angora, Ltd. (Jiangsu, China). A total of 10 ferrets (*n* = 2 per group) were anesthetized with ketamine (20 mg/kg, Hospira, Melbourne, Australia) and xylazine (1 mg/kg, Merck, Darmstadt, Germany) and i.n. inoculated with 10^6^ TCID_50_ of the test virus in 0.5 mL volume. All ferrets in each group were monitored daily for body temperature. The ferrets were subsequently euthanized on 4 dpi, and nasal turbinate, trachea, six lung lobes, spleen, kidney, liver, and brain samples were collected for virus titration. Tissues of right lung lobes were also used for histology and IHC.

### Ferret transmission experiments

A total of 54 ferrets (*n* = 9 per virus group) were used in DC and RD transmission experiments. One ferret was anesthetized and i.n. inoculated with 10^6^ TCID_50_ of each virus in 0.5 mL volume. On the next day, the inoculated ferret was individually paired by cohousing with a DC ferret; an RD-contact ferret was also housed in a wire frame cage adjacent to the infected ferret. The infected and RD-contact ferrets were 5 cm apart. Three independent replicates were conducted for each virus group. To monitor virus shedding, nasal washes were collected from all ferrets every other day for 14 days. At the same days, air sampling was collected using aerosol particle liquid concentrator (Portable Bioaerosol Sampler WA-15, Beijing Dingblue Technology Co., Ltd., Beijing, China) (39). Air was drawn for 40 min at a flow rate of 15 L per minute (total volume of 600 L) in contact with 3 mL sampling fluid. After the sampling, the remaining volume of the collection fluid dropped to about 1.5–2 mL due to evaporation. From each sample, 200 µL was taken for subsequent RNA isolation. From the remaining sample, 200 µL was individually inoculated into three 10-day-old embryonated chicken eggs. Infectious virus in the air samples was determined by specific pathogen-free chicken embryos. The air samples were determined to be positive if at least one allantoic fluid of the inoculated embryos was positive in HA assay. The HA assay result of each chicken embryo is presented as an independent record. Ferrets that lost >30% of their initial body weight were euthanized. Sera were collected from all ferrets at 21 dpi, and seroconversion was analyzed by HI assay.

### RNA isolation and qRT-PCR

RNA isolation from the air samples was performed using a fully automated nucleic acid extraction and purification instrument (Zhijie Biotechnology Co., Ltd., Henan, China). After isolation, the RNA was kept on an ice block, and 5 µL of it was transferred to a new plate with qRT-PCR mix containing primers and probe mix targeting the M gene of influenza A viruses, as well as Neoscript Fast RT Premix-UNG (Zhuhai Biori Biotechnology Co., Ltd., Guangdong, China) and PCR-grade water. The cutoff threshold was set manually after examination of the background signals and the negative control. Samples with a cycle threshold (Ct) of 40 and above were considered negative. RNA copy numbers were extrapolated using a standard curve based on samples of known M gene copy numbers.

### Polymerase activity assay

Viral polymerase activity was determined using a luciferase reporter assay as previously described (20). The PB2, PB1, NP, and PA genes from H3 viruses were separately cloned into the pcDNA3.1(+) plasmid. HEK293T cells were co-transfected with PB2, PB1, NP, and PA expression plasmids (125 ng), together with the firefly luciferase reporter plasmid p-Luci (10 ng), and internal control pRL-TK (2.5 ng) at the indicated temperature using JetPRIME (Polyplus, New York, NY, USA) according to the manufacturer’s instructions. At 24 h post-transfection, cell lysates were prepared using the Dual-Luciferase Reporter Assay System (Promega, Madison, WI, USA), and the luciferase activities were measured on a GloMax 96 microplate luminometer.

### Statistical analyses

All statistical analyses were performed using Prism V.8.0.2 (GraphPad Software). Statistical significance was assessed using Student’s *t*-test, one-way analysis of variance, followed by a Dunnett’s *post hoc* test, or a chi-squared test. *P* < 0.05 was considered to indicate a statistically significant difference.

## Data Availability

The sequences generated in this study have been deposited in the GenBank database, and the accession numbers are listed in Table S6.

## SUPPLEMENTAL MATERIAL

Supplemental material mbio.00677-25-s0001.docx Fig. S1 to S4; Tables S1 to S3; captions for Tables S4 to S6.

Table S4 mbio.00677-25-s0002.xlsx Specific amino acid substitutions in H3Ny viruses.

Table S5 mbio.00677-25-s0003.xlsx Key molecular markers of the H3Ny viruses.

Table S6 mbio.00677-25-s0004.xlsx Accession numbers for the new sequences generated in this study.

ASM does not own the copyrights to Supplemental Material that may be linked to, or accessed through, an article. The authors have granted ASM a non-exclusive, world-wide license to publish the Supplemental Material files. Please contact the corresponding author directly for reuse.
