# Supplementary material for: Assessment of the public health risk of novel reassortant H3N3 avian influenza viruses that emerged in chickens
Source: mBio. 2025 Jun 16;16(7):e00677-25. doi: 10.1128/mbio.00677-25 (PMC12239568; doi:10.1128/mbio.00677-25)
Supplement: Supplemental material — Fig. S1 to S4; Tables S1 to S3; captions for Tables S4 to S6. [file mbio.00677-25-s0001.docx]

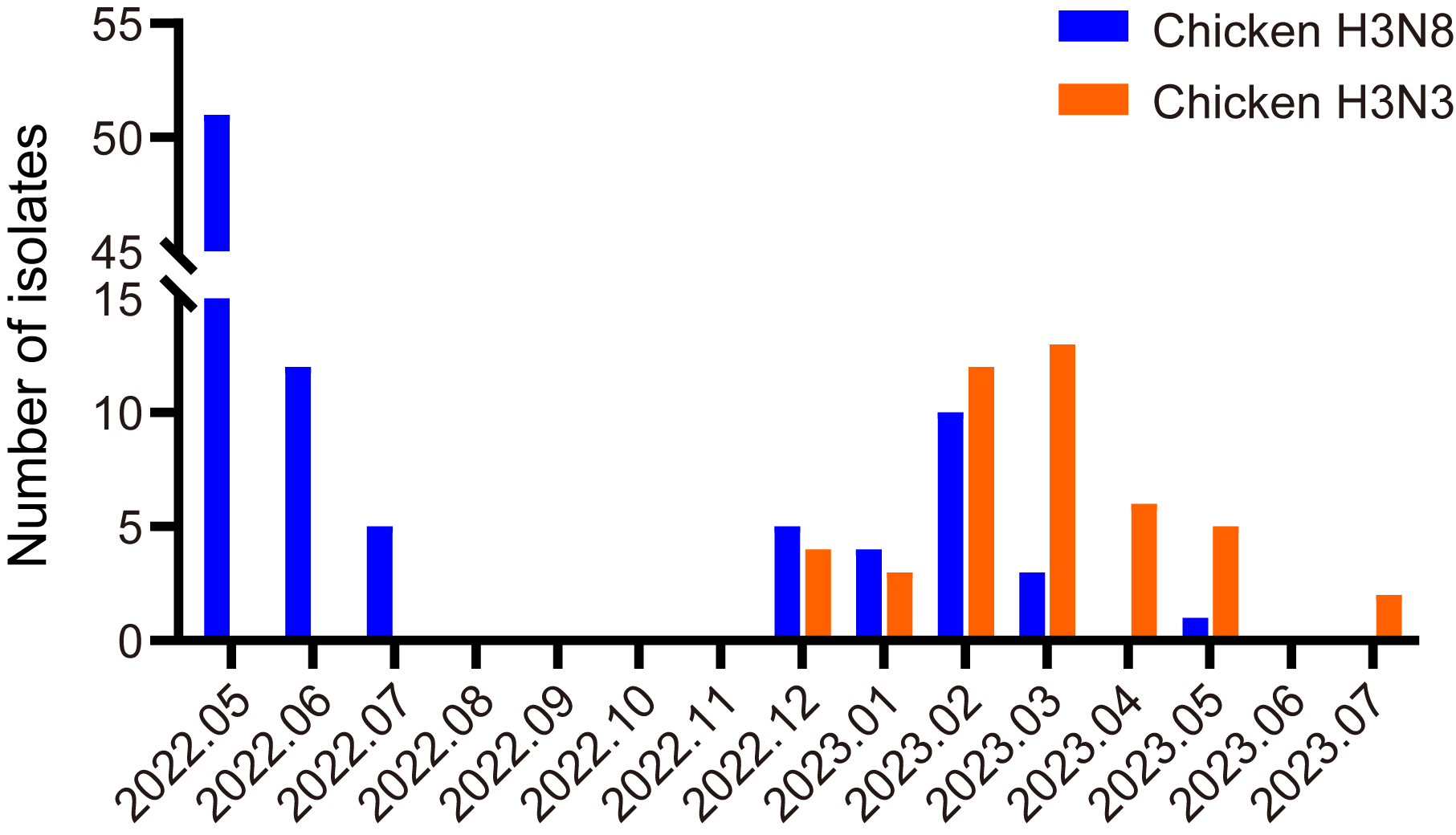
**Supplementary Figures**


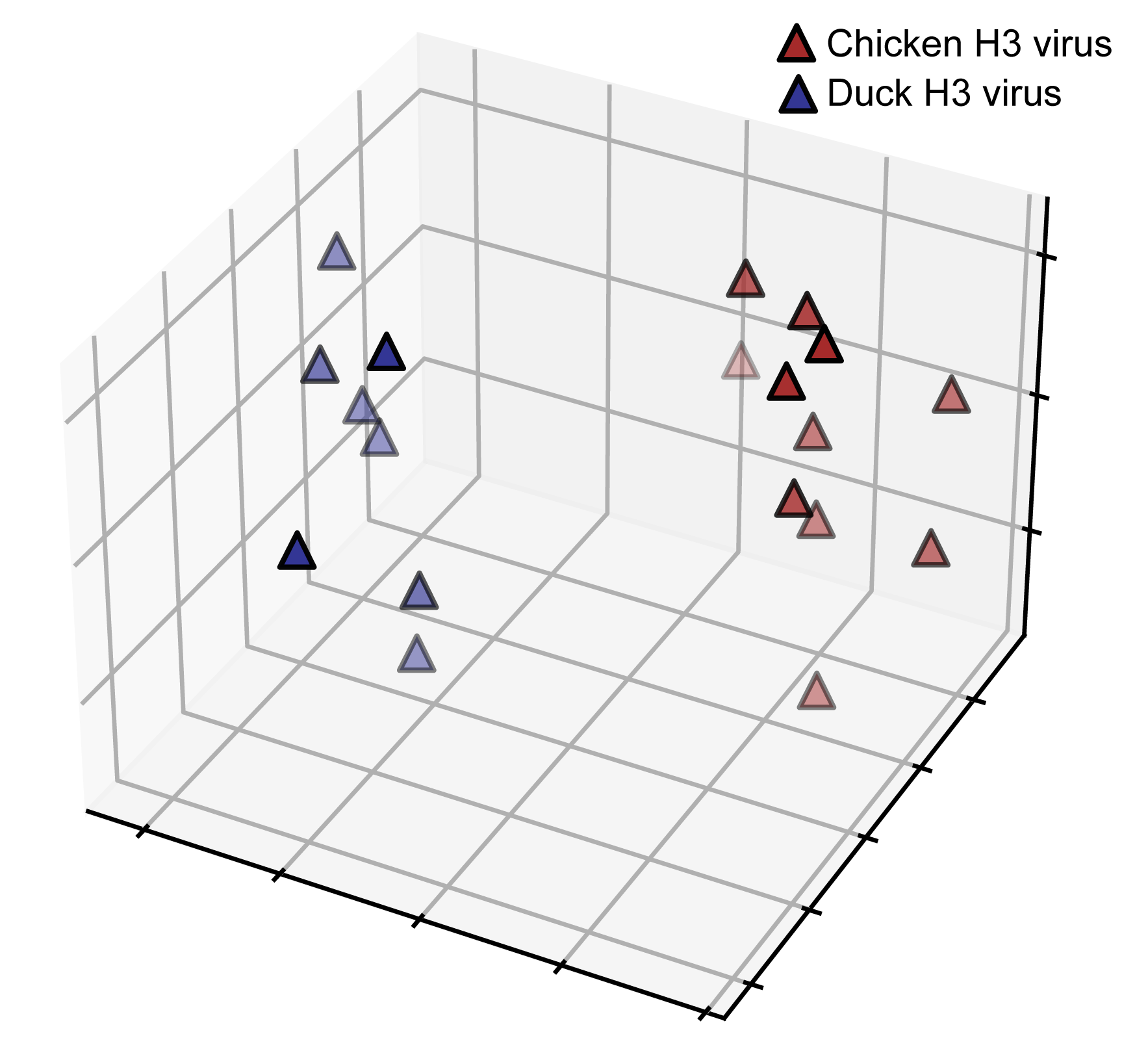
**Fig. S1. Isolation number of H3 subtype AIVs in the period of 2022.05-2023.07 from chicken.**

**Fig. S2. Antigenic map based on the HI assay data.** Antigenic map was drawn by performing multidimensional scaling (MDS) with downscaling of the HI data to three-dimensional space after log2-transformation, using K-means clustering. The vertical and horizontal axes both represent antigenic distance. The spacing between grid lines is 1 unit of antigenic distance, corresponding to a twofold dilution of antiserum in the HI assay. The details of the HI data were shown in Supplementary Table 2.


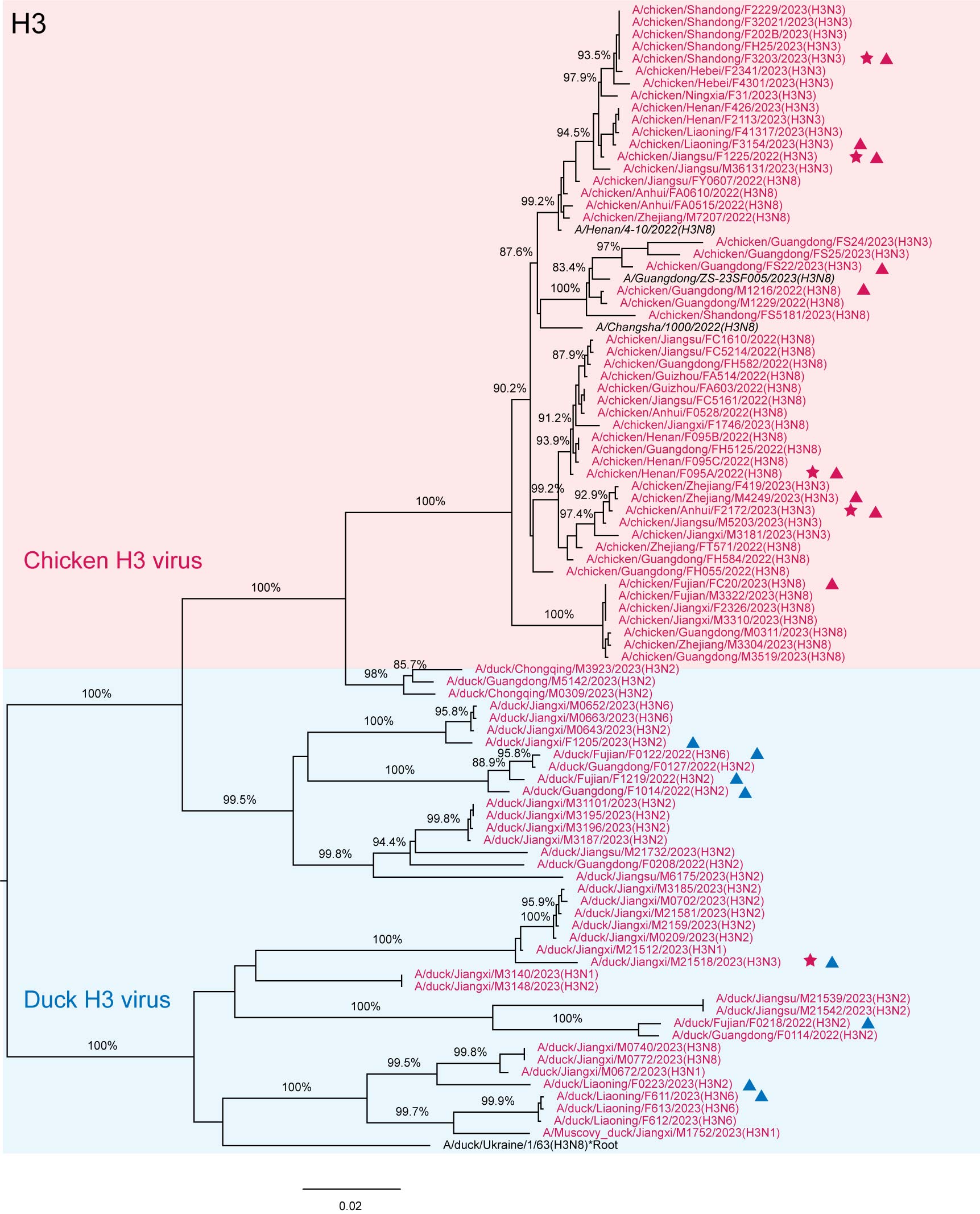


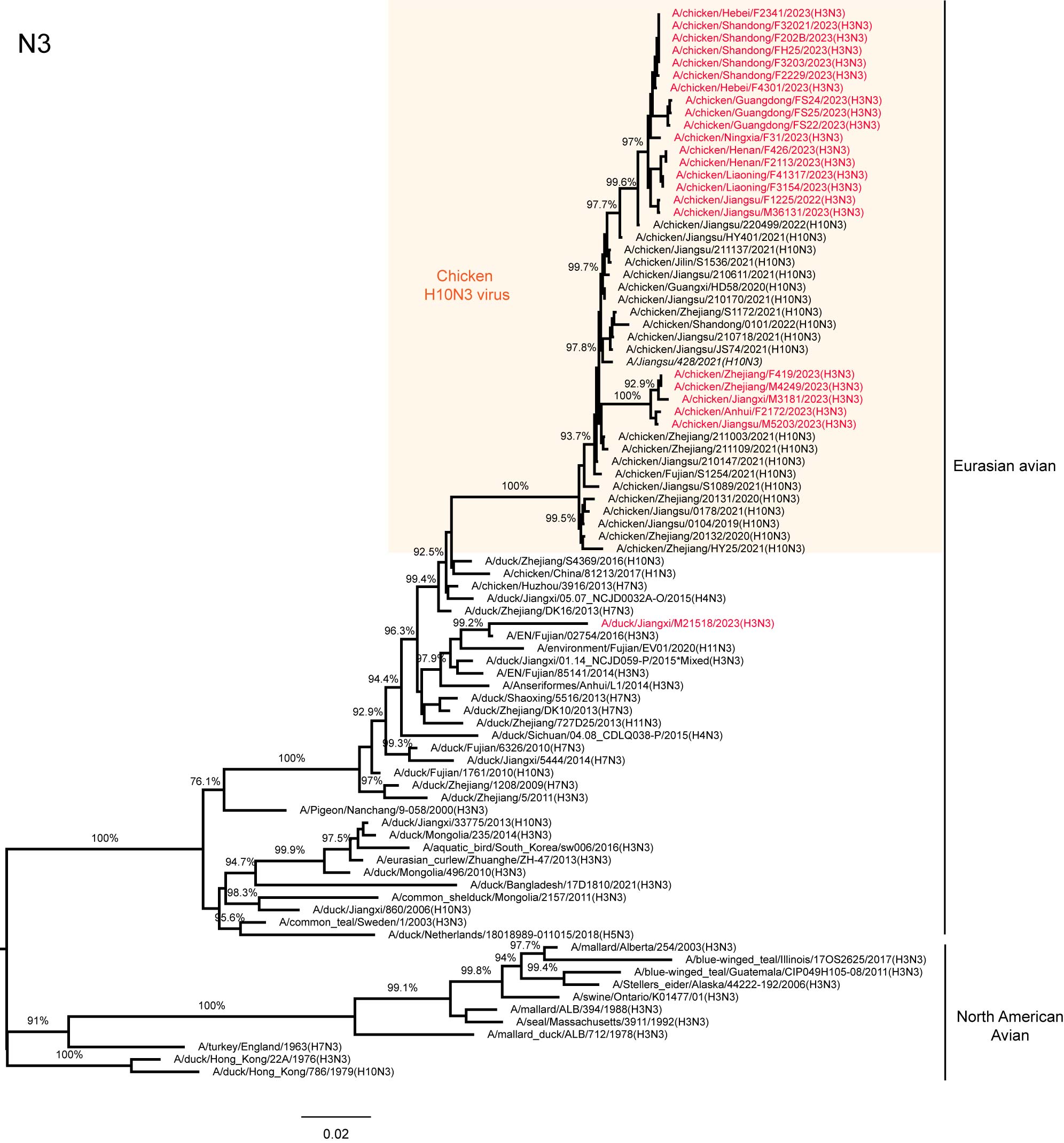


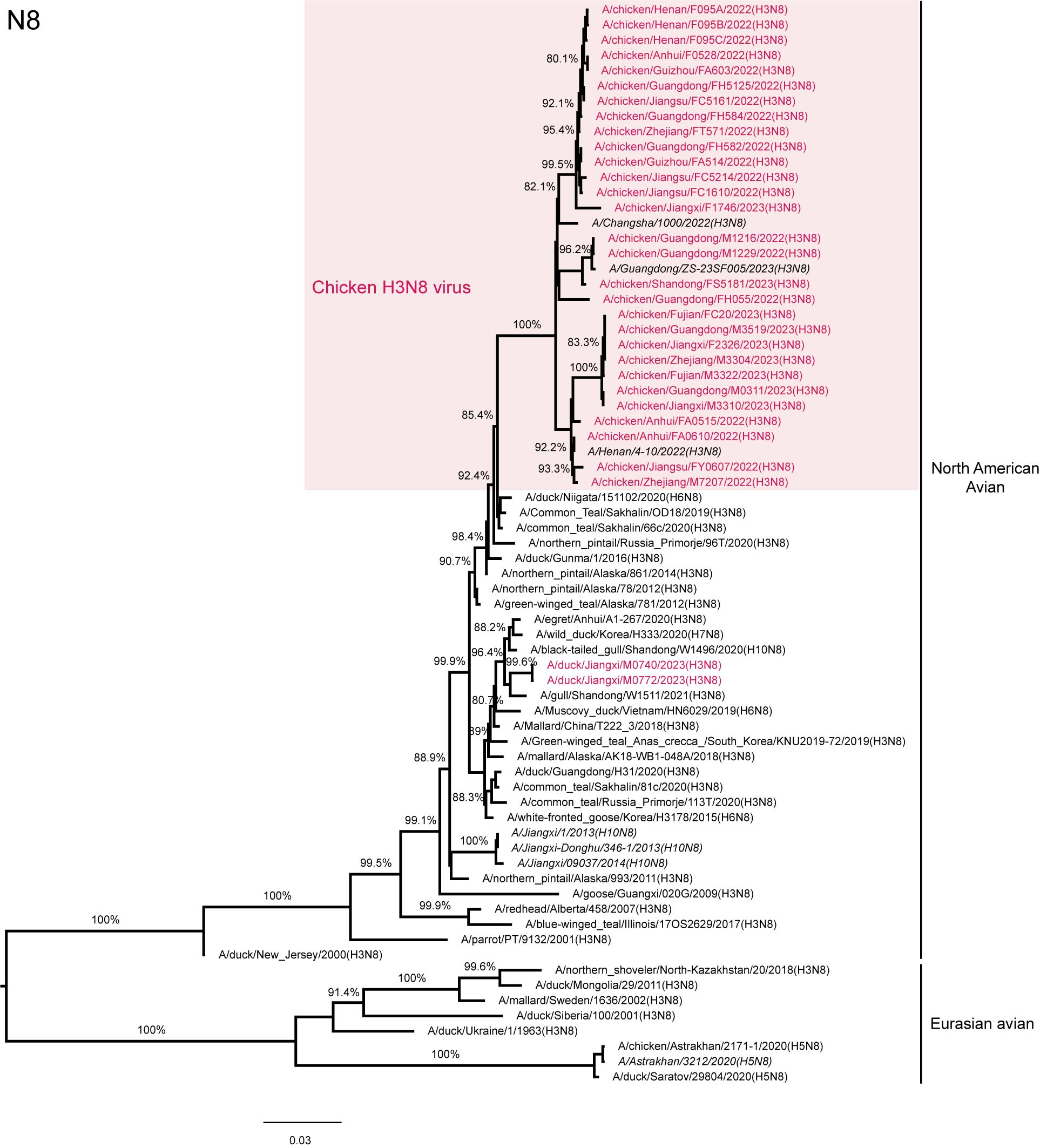


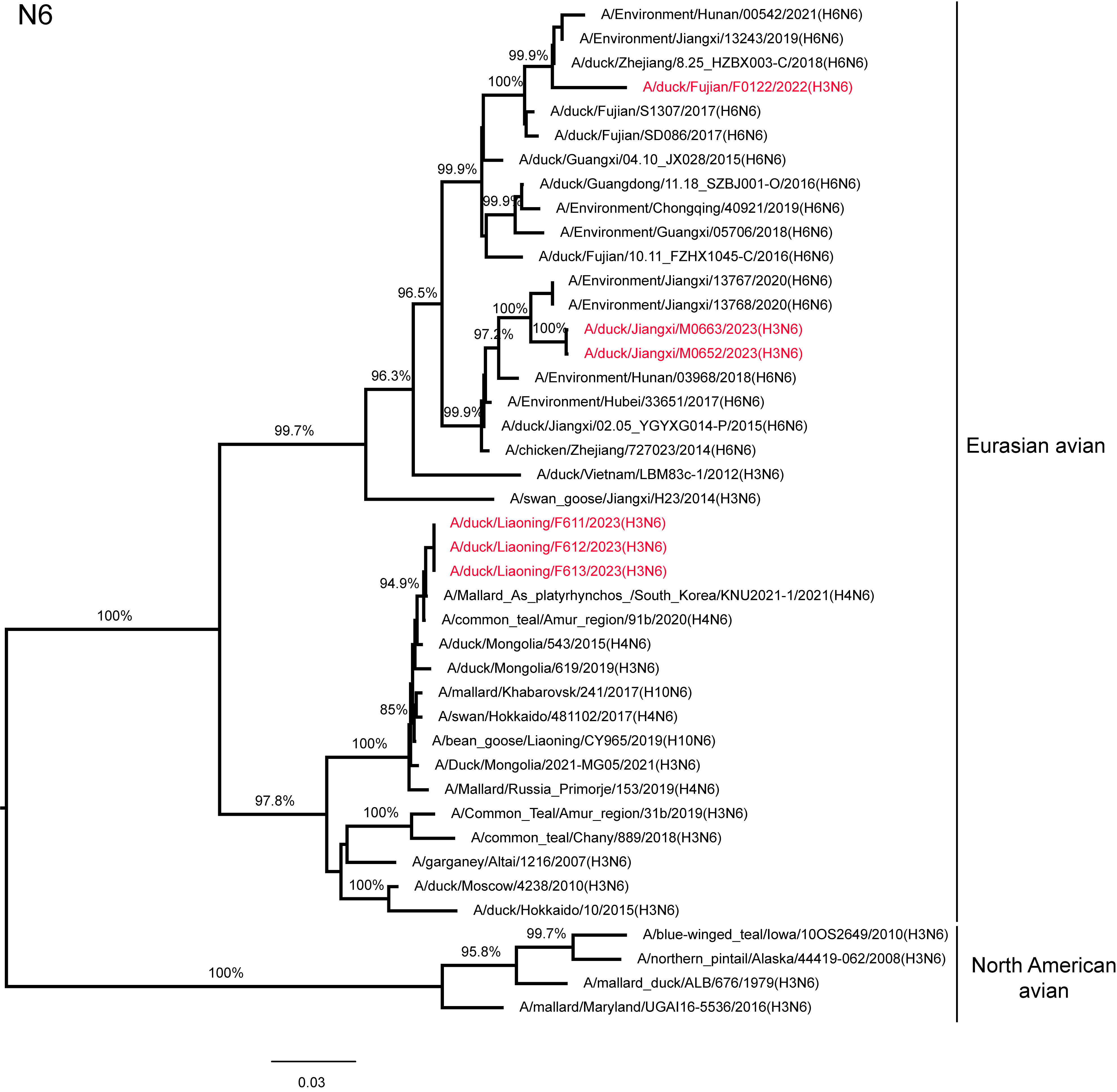


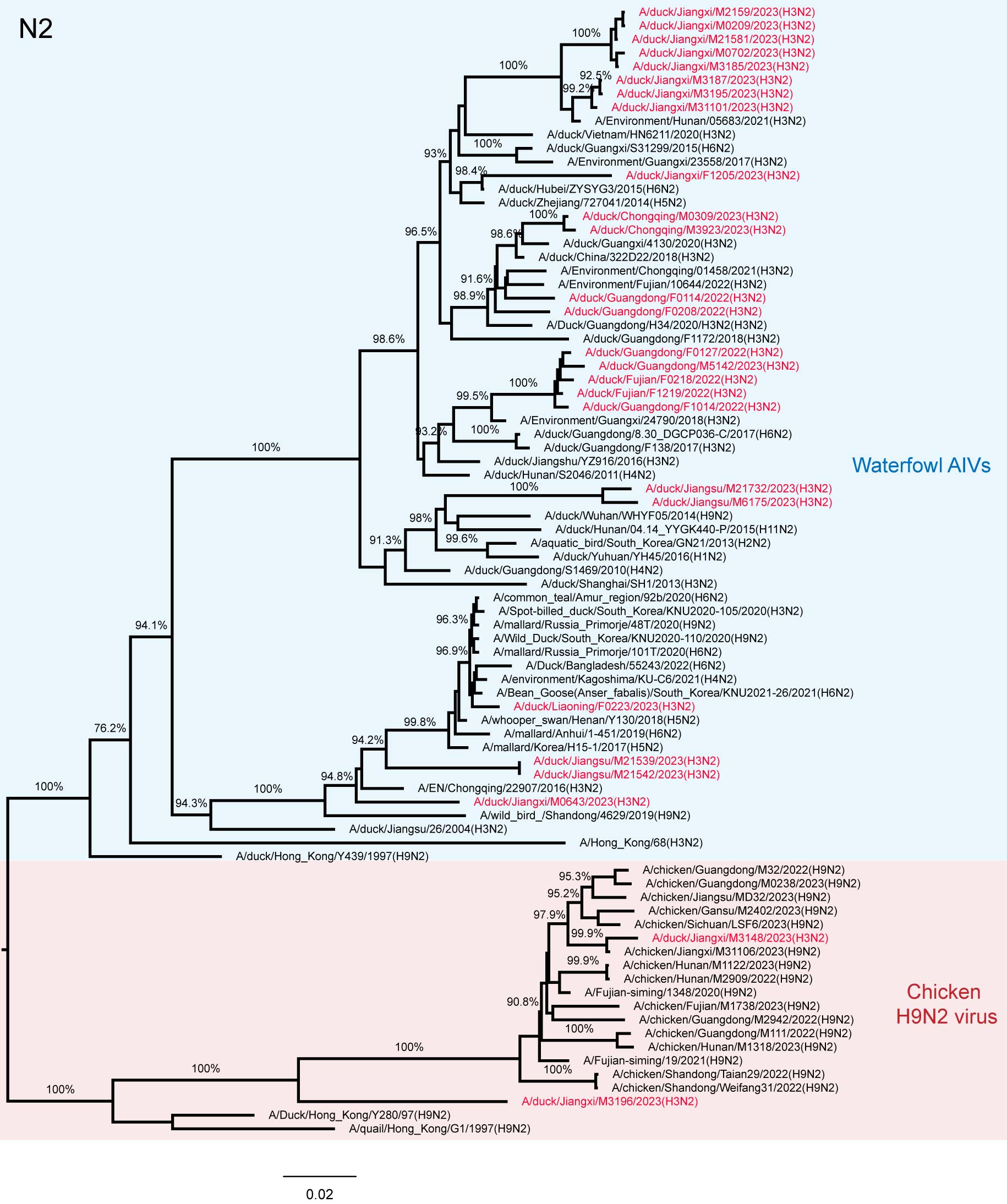


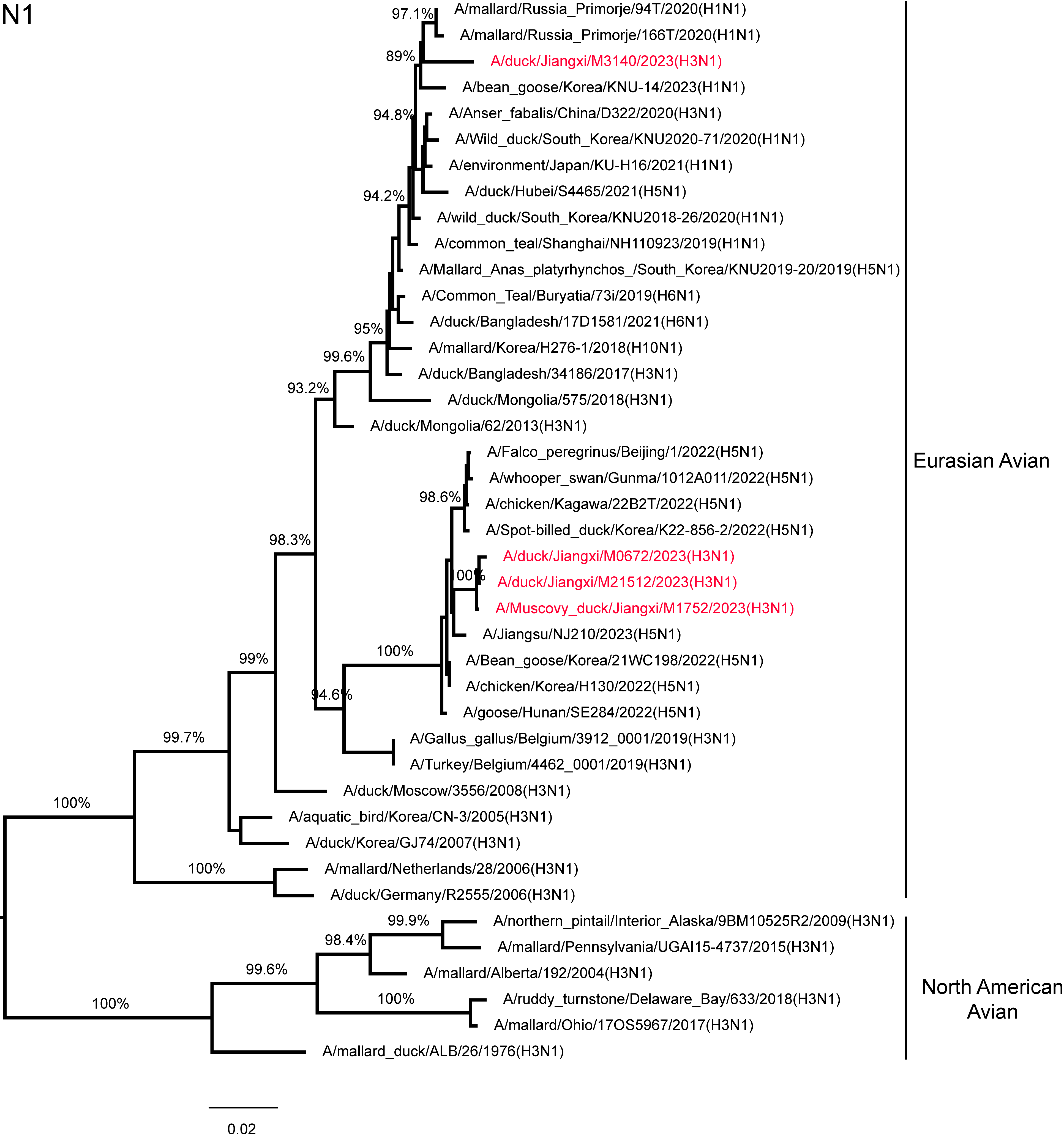


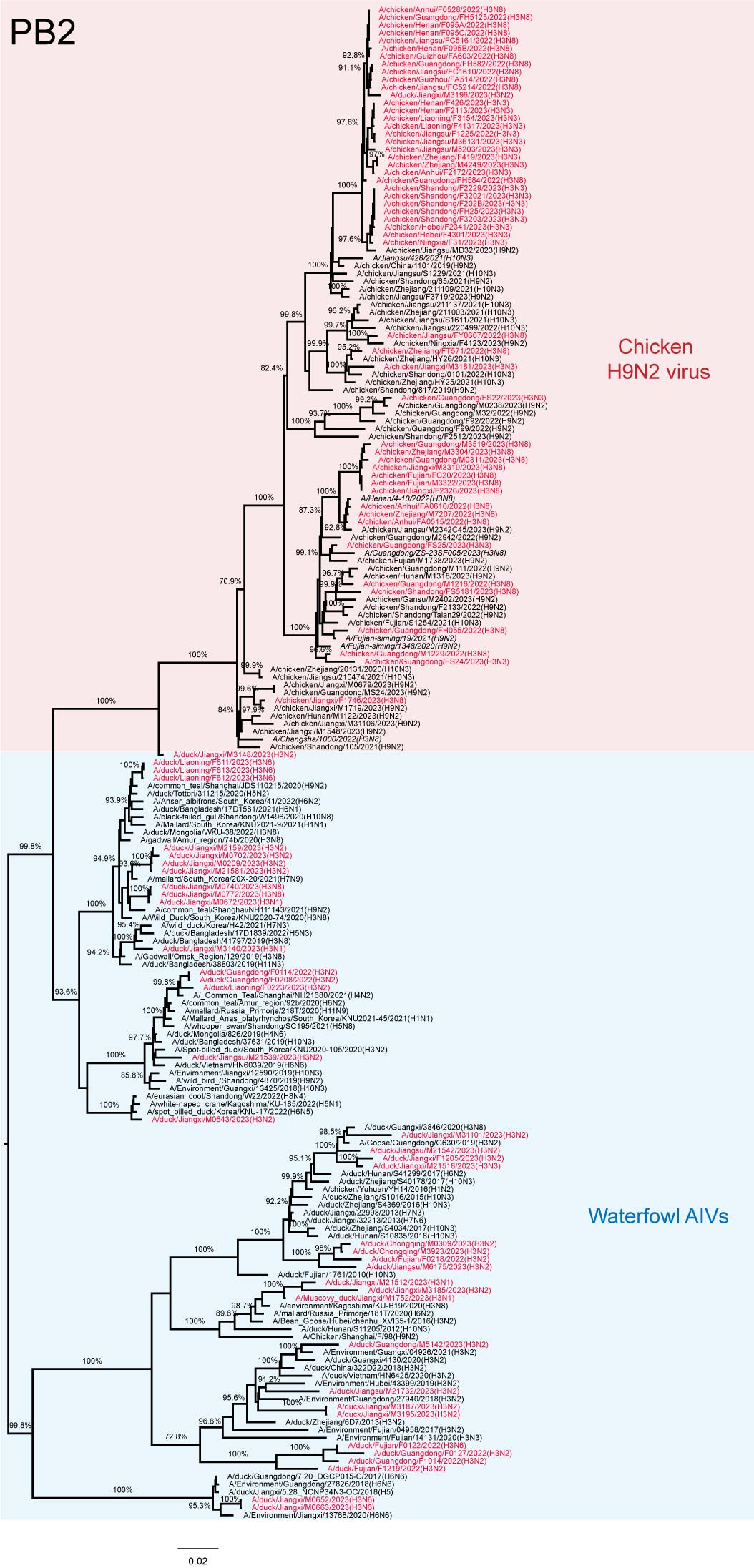


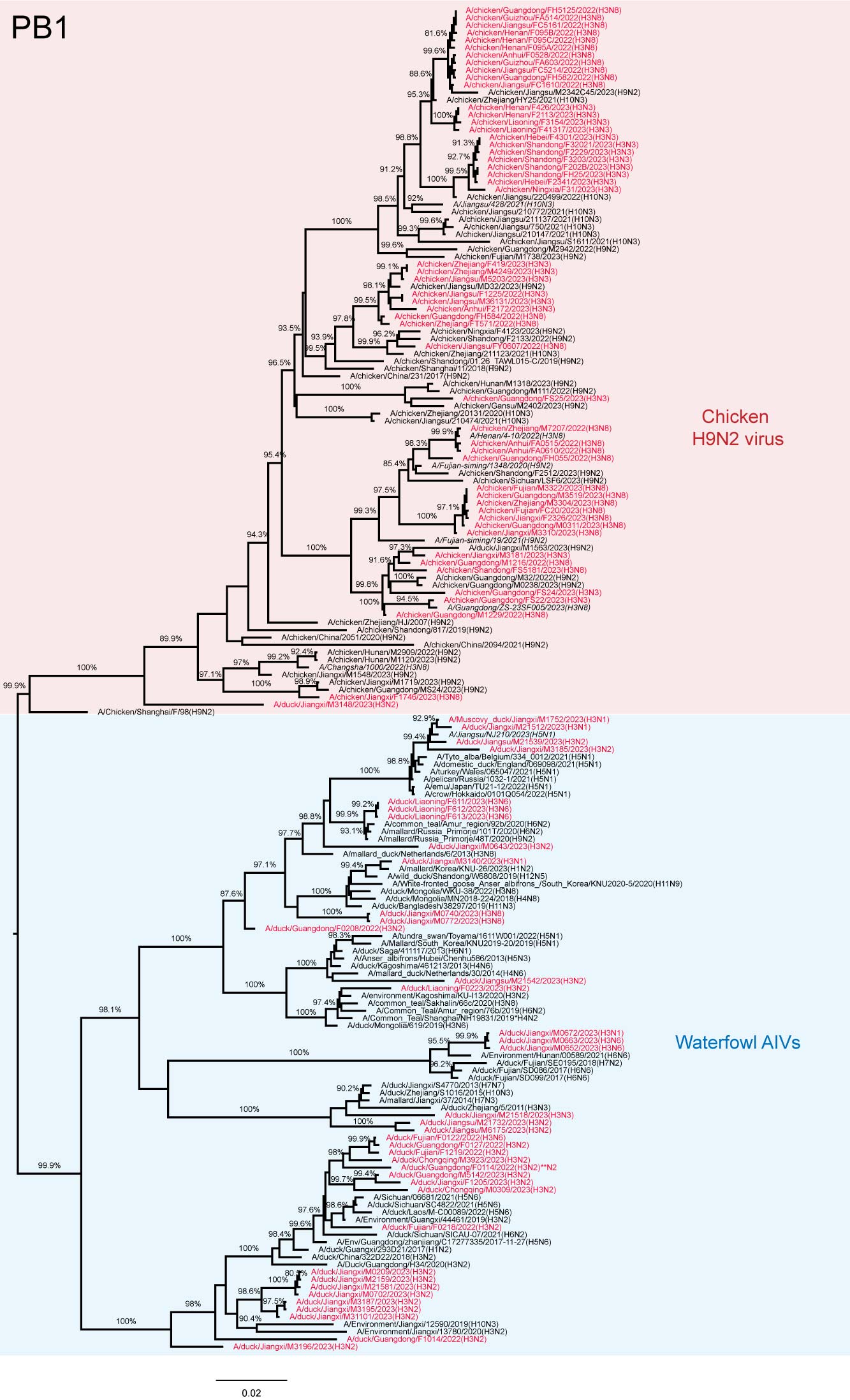


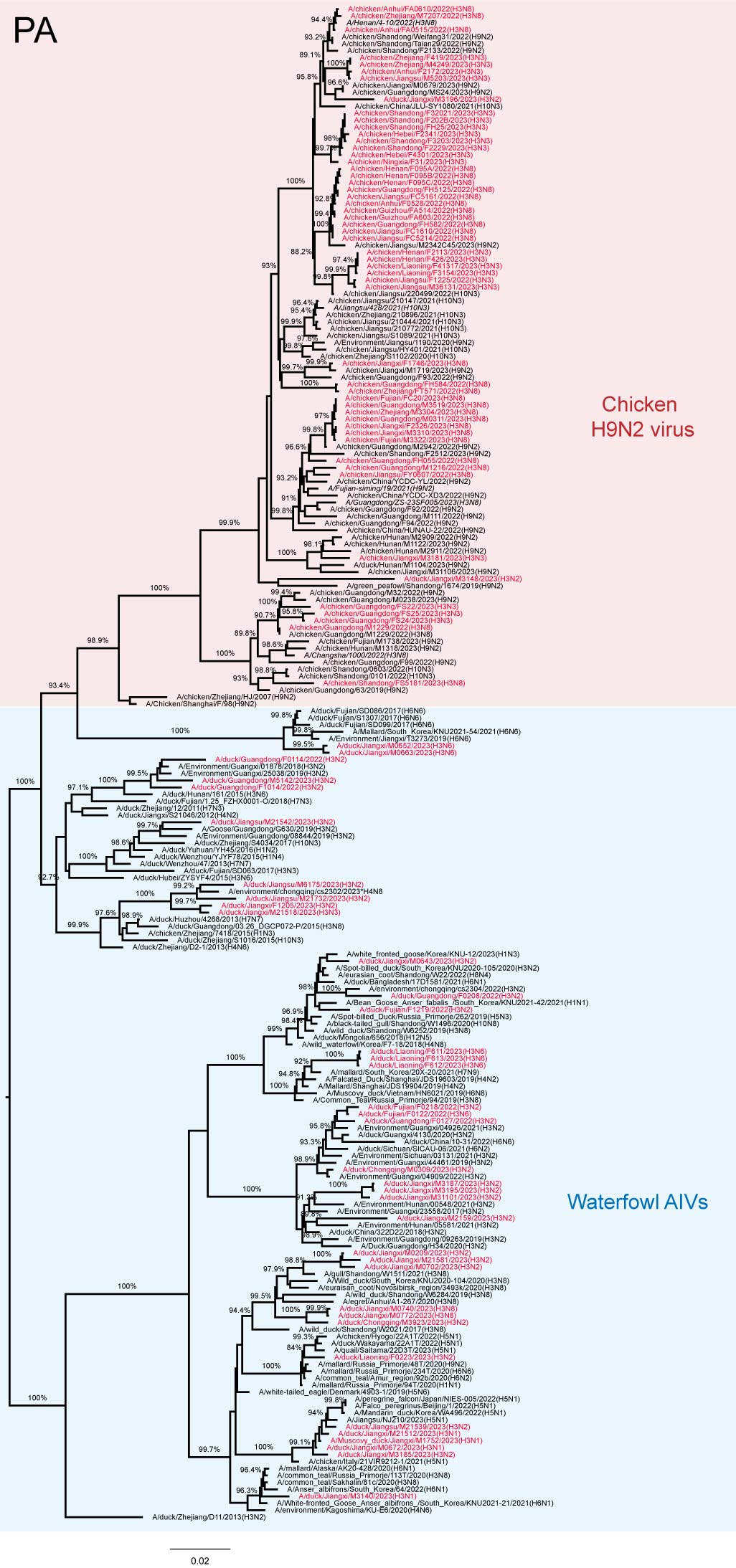


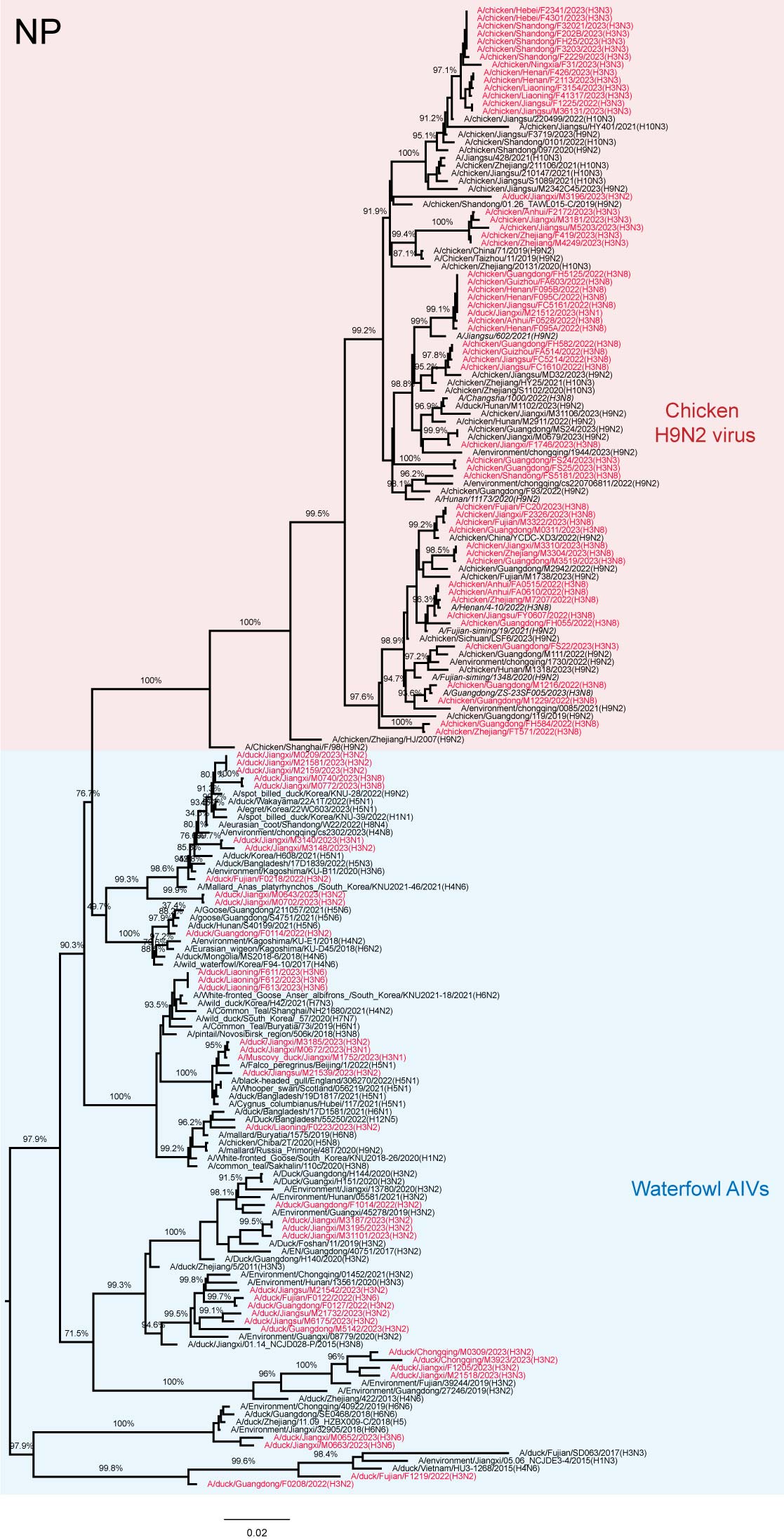


**
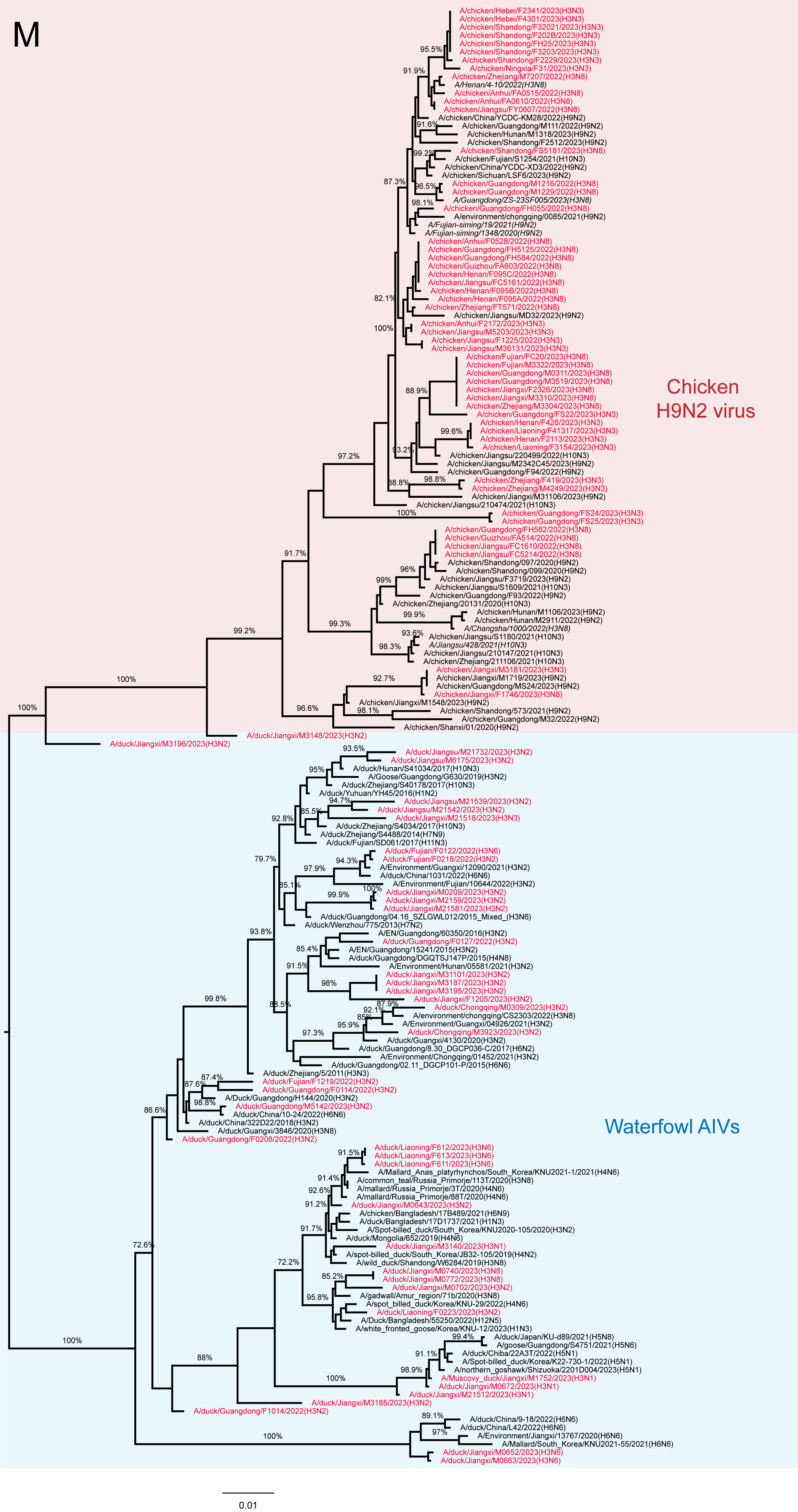
**


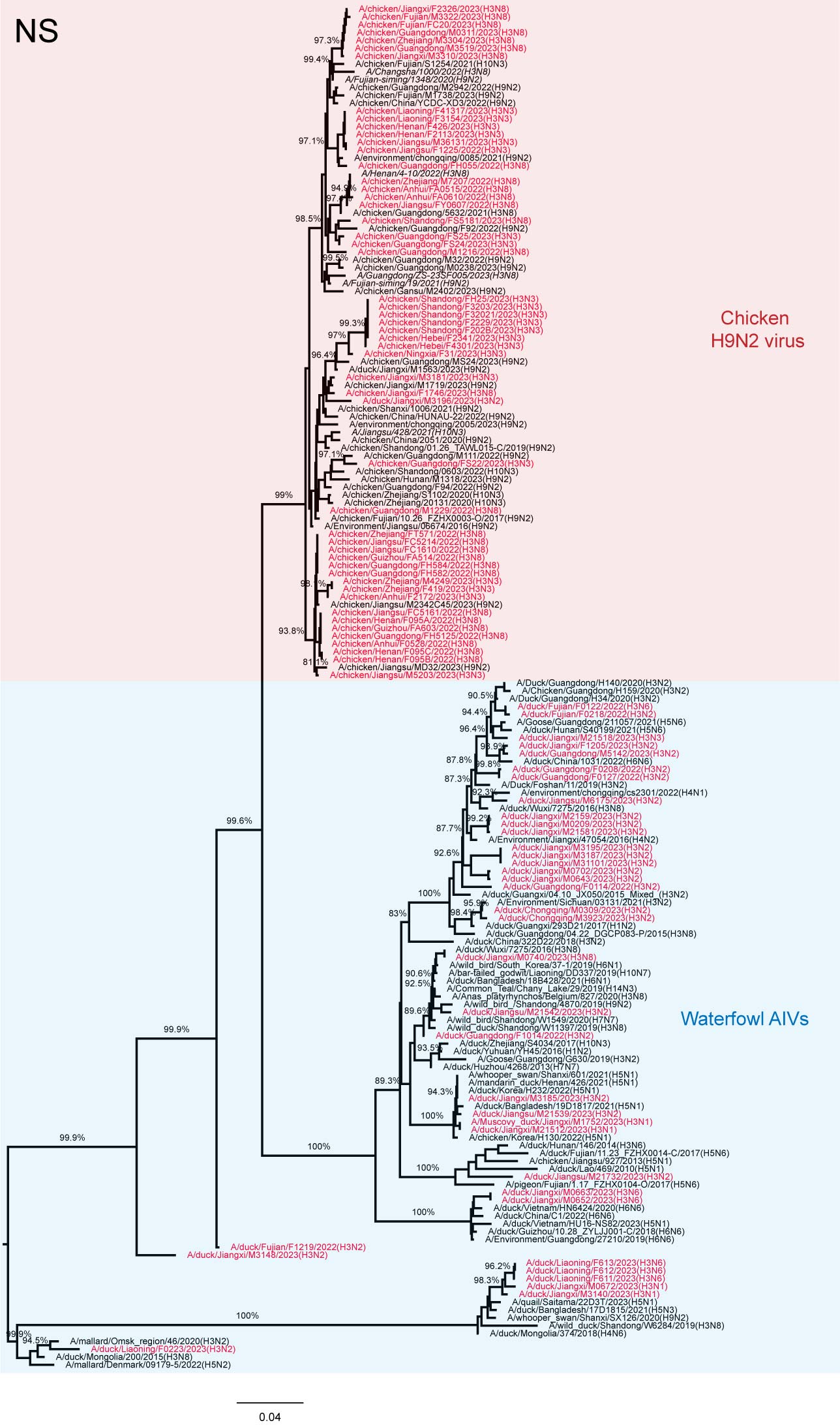


**Fig. S3. Phylogenetic relationships of fully sequenced H3 subtype AIVs.** The phylogenetic trees were generated by maximum likelihood methods. H3 viruses isolated in this study were colored red. Sequences of viruses with names in black were downloaded from public databases. Lineages are labelled with lines on the right. The strains used for biological characterization were indicated by asterisks. The strains used for antigenic analyses were indicated by triangles. Scale bar is in units of nucleotide substitutions per site. Node labels represent bootstrap values.


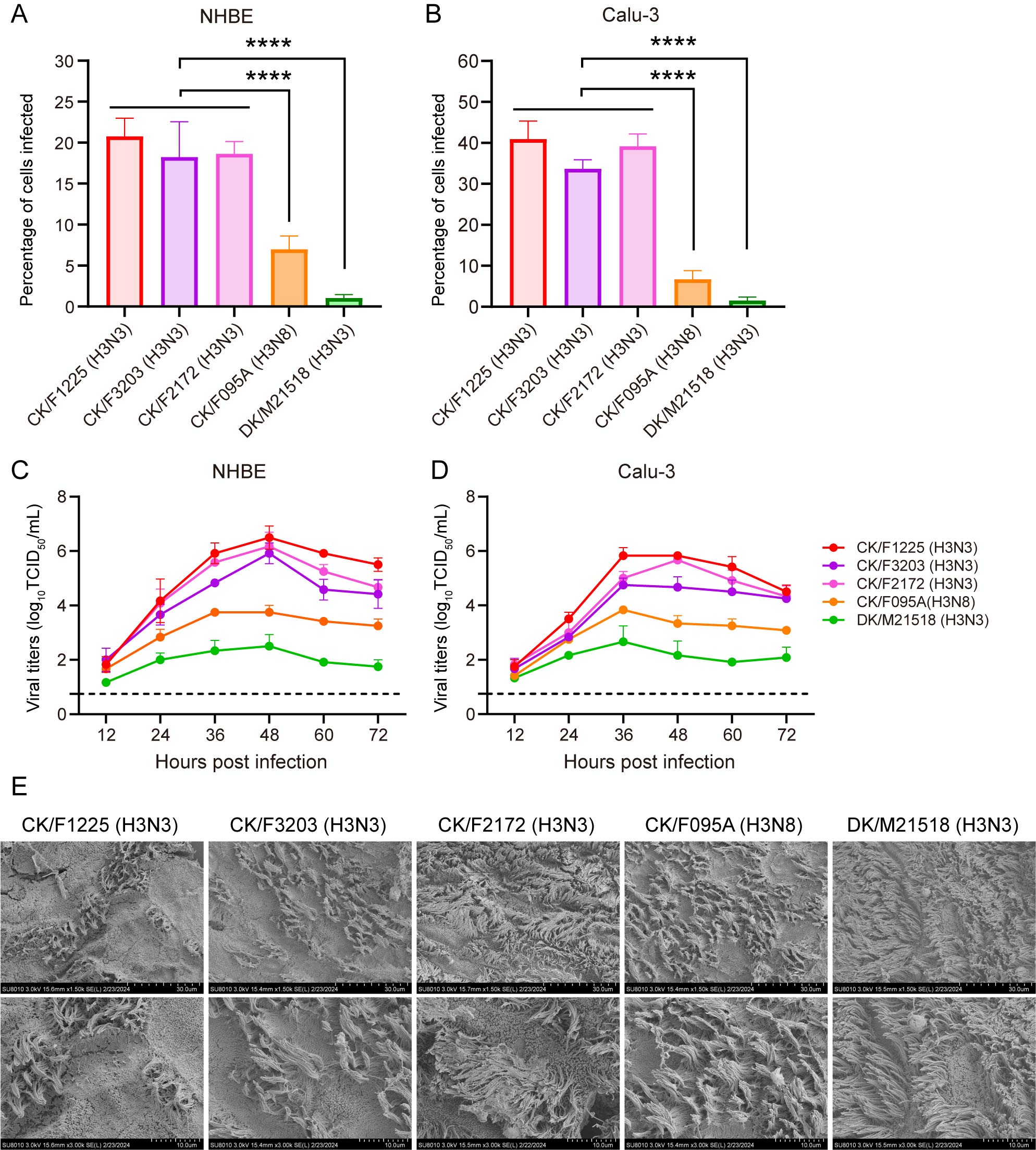
**Fig. S4. Infection and replication of H3 AIVs in NHBE and Calu-3 cells. A** and **B.** NHBE and Calu-3 cells were infected with the indicated viruses at an MOI of 1.0. Influenza virus NP was detected by indirect immunofluorescence. Nuclei were detected with DAPI. Viral NP expression was quantified in viruses-infected NHBE (A) and Calu-3 (B) cells. Percentages of influenza NP-positive cells per DAPI-positive cells were calculated. Values are expressed as mean ± SD of three randomly selected fields. **C** and **D**. Growth kinetics of H3 viruses were ascertained in well-differentiated NHBE cultures (C) and Calu-3 cells (D) infected with an MOI of 0.01. At indicated time points, cell culture supernatants were collected and virus titers were determined by TCID_50_ assays on MDCK cells. **E.** Cytopathic effects of H3 viruses infection in NHBE cells. NHBE cells were infected with the indicated viruses at an MOI of 0.01. Scanning electron microscopy was used to detect changes in the morphology of NHBE cultures at 48 hpi. Scale bar above represents 30 μm; scale bar below represents 10 μm.

**Supplementary Tables**

**Table S1. Isolation of H3 subtype viruses in poultry from 2022.5-2023.7**

| **Table1. Isolation of H3 subtype viruses in poultry from 2022.5-2023.7** | | | | | | | | |
| --- | --- | --- | --- | --- | --- | --- | --- | --- |
| **Location** | **Host** | **Number of samples collected** | **Number of AIV positive samples** | **Number of H3Ny virus isolates** | | | | |
|  |  |  |  | **Total** | **Subtype** | **Number of isolates** | **Number of deep seq.** | **Provinces found with H3Ny virus** |
| Market | chicken | 3184 | 421 | 42 | H3N3 | 9 | 4 | Fujian, Jiangsu, Jiangxi |
|  |  |  |  |  | H3N8 | 26 | 8 | Guangdong, Fujian, Jiangxi, Zhejiang |
|  | duck | 982 | 279 | 64 | H3N1 | 7 | 4 | Jiangxi |
|  |  |  |  |  | H3N2 | 45 | 18 | Guangdong, Fujian, Jiangxi, Jiangsu, Chongqing |
|  |  |  |  |  | H3N3 | 2 | 1 | Jiangxi |
|  |  |  |  |  | H3N6 | 3 | 2 | Jiangxi |
|  |  |  |  |  | H3N8 | 7 | 2 | Jiangxi |
| Farm | chicken | 634 | 302 | 94 | H3N3 | 36 | 18 | Anhui, Guangdong, Hebei, Henan, Jiangsu, Liaoning, Ningxia, Shandong, Zhejiang |
|  |  |  |  |  | H3N8 | 65 | 21 | Anhui, Fujian, Guangdong, Guizhou, Henan, Jiangsu, Jiangxi, Shandong, Zhejiang |
|  | duck | 65 | 32 | 12 | H3N6 | 4 | 4 | Liaoning, Fujian |
|  |  |  |  |  | H3N2 | 8 | 8 | Liaoning, Fujian, Jiangxi, Guangdong |

**Table S2. Antigenic classification of H3 subtype AIVs by HI assays**

| **Antisera** | | | | | | | | | | |
| --- | --- | --- | --- | --- | --- | --- | --- | --- | --- | --- |
| **Virus** | **A/CK/JS/F1225/2022(H3N3)** | **A/CK/SD/F3203/2023(H3N3)** | **A/CK/AH/F2172/2023(H3N3)** | **A/CK/HN/F095A/2022(H3N8)** | **A/CK/GD/F0103/2022(H3N8)** | **A/CK/FJ/FC20/2023(H3N8)** | **A/DK/JX/M21518/2023(H3N3)** | **A/DK/FJ/F0122/2022(H3N6)** | **A/DK/LN/F611/2023(H3N6)** | **A/DK/LN/F0223/2023(H3N2)** |
| **A/CK/JS/F1225/2022(H3N3)** | 256 | 512 | 256 | 128 | 64 | 64 | 32 | 16 | 8 | 8 |
| **A/CK/SD/F3203/2023(H3N3)** | 256 | 256 | 256 | 128 | 32 | 256 | 64 | 32 | 8 | 16 |
| **A/CK/AH/F2172/2023(H3N3)** | 128 | 256 | 128 | 128 | 32 | 256 | 64 | 16 | 8 | 8 |
| **A/CK/NC/F3154/2023(H3N3)** | 128 | 128 | 128 | 128 | 64 | 128 | 32 | 16 | 4 | 8 |
| **A/CK/GD/FS22/2023(H3N3)** | 128 | 256 | 128 | 128 | 32 | 256 | 32 | 16 | 8 | 8 |
| **A/CK/ZJ/M4249/2023(H3N3)** | 256 | 256 | 256 | 128 | 64 | 256 | 64 | 16 | 4 | 16 |
| **A/CK/HN/F095A/2022(H3N8)** | 256 | 256 | 256 | 256 | 128 | 512 | 32 | 16 | 4 | 8 |
| **A/CK/AH/FE12/2022(H3N8)** | 256 | 512 | 256 | 256 | 128 | 512 | 64 | 32 | 8 | 16 |
| **A/CK/GD/F0103/2022(H3N8)** | 256 | 256 | 256 | 128 | 128 | 256 | 64 | 32 | 8 | 16 |
| **A/CK/GD/M1216/2022(H3N8)** | 128 | 128 | 128 | 128 | 64 | 256 | 32 | 16 | 8 | 8 |
| **A/CK/FJ/FC20/2023(H3N8)** | 256 | 256 | 256 | 256 | 128 | 256 | 64 | 64 | 16 | 64 |
| **A/DK/JX/21518/2023(H3N3)** | 32 | 32 | 32 | 32 | 16 | 32 | 128 | 32 | 8 | 64 |
| **A/DK/FJ/F0122/2022(H3N6)** | 128 | 128 | 128 | 32 | 32 | 8 | 256 | 32 | 8 | 16 |
| **A/DK/LN/611/2023(H3N6)** | 128 | 128 | 128 | 32 | 32 | 32 | 512 | 32 | 64 | 16 |
| **A/DK/LN/F0223/2023(H3N2)** | 32 | 32 | 32 | 16 | 8 | 32 | 256 | 64 | 32 | 128 |
| **A/DK/GD/F0114/2022(H3N2)** | 128 | 64 | 128 | 32 | 64 | 32 | 512 | 64 | 16 | 32 |
| **A/DK/JX/F1205/2023(H3N2)** | 128 | 128 | 128 | 64 | 16 | 16 | 512 | 32 | 8 | 16 |
| **A/DK/FJ/F0218/2022(H3N2)** | 64 | 32 | 64 | 64 | 32 | 16 | 512 | 32 | 8 | 16 |
| **A/DK/FJ/F1219/2022(H3N2)** | 64 | 64 | 128 | 64 | 16 | 8 | 256 | 32 | 4 | 8 |

**Table S3. Virulence and transmission of H3 viruses in ferrets.**

| Virus | Inoculated ferrets | | Contact ferrets | | Aerosol ferrets | |
| --- | --- | --- | --- | --- | --- | --- |
|  | Maximum weight loss (%) | Seroconversion: positive/total  (HI titer) | Maximum weight loss (%) | Seroconversion: positive/total  (HI titer) | Maximum weight loss (%) | Seroconversion: positive/total  (HI titer) |
| BJ/0212  (H1N1) | 6.15 | 3/3 (640,640,640) | 5.88 | 3/3  (640, 640, 640) | 5.50 | 3/3(640,640,640) |
| CK/F1225  (H3N3) | 4.80 | 3/3 (160,160,160) | 5.29 | 3/3  (160, 80, 160) | - | 0/3 |
| CK/F3203  (H3N3) | 4.92 | 3/3 (160,160,160) | 4.82 | 3/3  (160, 80, 80) | - | 0/3 |
| CK/F2172  (H3N3) | 4.65 | 3/3 (160,160,160) | 4.94 | 3/3  (160, 80, 80) | - | 0/3 |
| CK/F095A  (H3N8) | 2.81 | 3/3 (160,160,160) | - | 0/3 | - | 0/3 |
| DK/M21518  (H3N3) | 1.35 | 3/3 (160,160,80) | - | 0/3 | - | 0/3 |

**Table S4. Specific amino acid substitutions in H3Ny viruses.** Key mutations were marked in red. '-': absence of amino acid residues.

**Table S5. List of Key molecular markers of the H3Ny viruses.** Key molecular markers of each virus were shown in Table S4.

**Table S6. Accession numbers for new sequences generated in this study.**
